# Septin binding restricts *Shigella* growth and triggers intracellular tolerance

**DOI:** 10.1073/pnas.2529720123

**Published:** 2026-10-27

**Authors:** Gizem Özbaykal Güler, Chen Zhang, Dominik Brokatzky, Rajdeep Das, Richard Wheeler, Ivo Gomperts Boneca, Teresa Cortes, Suliana Manley, Serge Mostowy

**Affiliations:** ^a^ Department of Infection Biology, London School of Hygiene and Tropical Medicine, London WC1E 7HT, United Kingdom; ^b^ Laboratory of Experimental Biophysics, School of Basic Sciences, Institute of Physics, Interfaculty Institute of Bioengineering, École Polytechnique Fédérale de Lausanne, Lausanne 1015, Switzerland; ^c^ Institut Pasteur, Université Paris Cité, CNRS Unité Mixe de Recherche 6047, INSERM U1306, Unité de Biologie et génétique de la paroi bactérienne, Paris F-75015, France; ^d^ Pathogen Gene Regulation Unit, Instituto de Biomedicina de Valencia, Consejo Superior de Investigaciones Científicas, Valencia 46010, Spain

**Keywords:** *Shigella*, bacterial growth, membrane potential, septins, host–pathogen interactions

## Abstract

Pathogen survival relies on their ability to adapt to changing environments, yet how intracellular bacteria tolerate cell-autonomous immunity remains unclear. Septins assemble into cage-like structures around cytoplasmic *Shigella* targeted by autophagy, but their antibacterial activity was unknown. Using advanced microscopy and cell-free reconstitution, we show that septins polymerize into circumferentially oriented filaments that inhibit bacterial growth. RNA-sequencing reveals that septin binding to *Shigella* upregulates type III secretion system (T3SS) effectors and triggers a stress response. Underlying this, septin-bound *Shigella* possess hyperpolarized membranes, but without lethally damaged cell envelope. We show that exposure to septin binding enhances the impact of the cell envelope-targeting antibiotic polymyxin B. During infection, T3SS effector OspC1 prevents septin recruitment, while stress-response regulator *cspC* protects against septin-mediated changes to membrane potential. Hence, septin binding is intrinsically antibacterial and is sensed by *Shigella* as a danger signal to promote mechanisms of intracellular tolerance.

## Significance

*Shigella*, the leading cause of bacillary dysentery, uses its virulence plasmid-encoded effectors to invade cells and manipulate host pathways for survival. Septins, cytoskeletal proteins known for roles in cell division, assemble into cage-like structures that entrap *Shigella*, yet antibacterial consequences of septin–bacteria interactions were not previously understood. We demonstrate septin binding to *Shigella* directly restricts bacterial growth, without assistance from canonical host defense pathways. Septin–*Shigella* interactions change bacterial gene expression, perturb voltage across the bacterial membrane, and activate a protective bacterial stress response. In agreement, *Shigella* have higher membrane potential and decreased intracellular survival during infection if this protective stress response is absent. Thus, septins do more than entrap cytosolic bacteria: They restrict growth by disrupting bacterial physiology.

The host cytoplasm is a hostile environment for invading bacteria due to defense mechanisms known as cell-autonomous immunity (1, 2). *Shigella*, a human-adapted and World Health Organization (WHO) high priority pathogen (3), can invade cells and spread within tissues by using effectors secreted by its type III secretion system (T3SS) (4, 5). *Shigella* T3SS effectors have been intensely investigated, enabling the discovery of several key cell-autonomous immunity pathways including antibacterial autophagy and its evasion by IcsB (6) and VirA (7), guanylate-binding protein (GBP) mediated immunity and its evasion by IpaH9.8 (8–10), as well as E3 ligase RNF213 mediated immunity and its evasion by IpaH1.4 (11–13). In contrast to evasion, mechanisms which promote tolerance to cell-autonomous immunity remain understudied.

Critical factors enabling invasive bacteria to survive and replicate during infection have been poorly understood, partly due to the highly heterogenous environment of the cytoplasm. For example, different subpopulations of bacteria can be targeted by distinct host defense mechanisms, such as GBPs or septin cage entrapment (14–17) creating multiple unique niches in which bacteria reside. Septins are cytoskeletal proteins best recognized for their role in cell division, membrane dynamics, and cell-autonomous immunity by acting as micron-scale curvature sensors (18, 19). In human cells, septins are found as hexamers and octamers with palindromic arrangement of two heterotrimers or two heterotetramers, respectively (20). Septin proteins are known to bind and polymerize on the plasma membranes of eukaryotic cells as well as synthetic membranes, where filaments can deform liposomes and induce membrane perturbations, suggesting a direct impact of septin binding on membrane structure (21–23). Understanding how septins interact with and impact membranes is central to septin function, and may have transformative implications for infection control.

In this work, we study *Shigella*–septin interactions to understand the antibacterial properties of septins and how cytoplasmic bacteria can respond to cell-autonomous immunity. We use in vitro reconstitution in combination with in situ infection models to reveal transcriptional and biophysical reprogramming of bacteria upon septin binding. Our findings highlight the intrinsic antibacterial activity of septins and identify a bacterial tolerance mechanism that enables survival in response to cell-autonomous immunity.

## Results

### Purified Septin Complexes Assemble Into Filaments on the *Shigella* Surface.

**Fig. 1.**
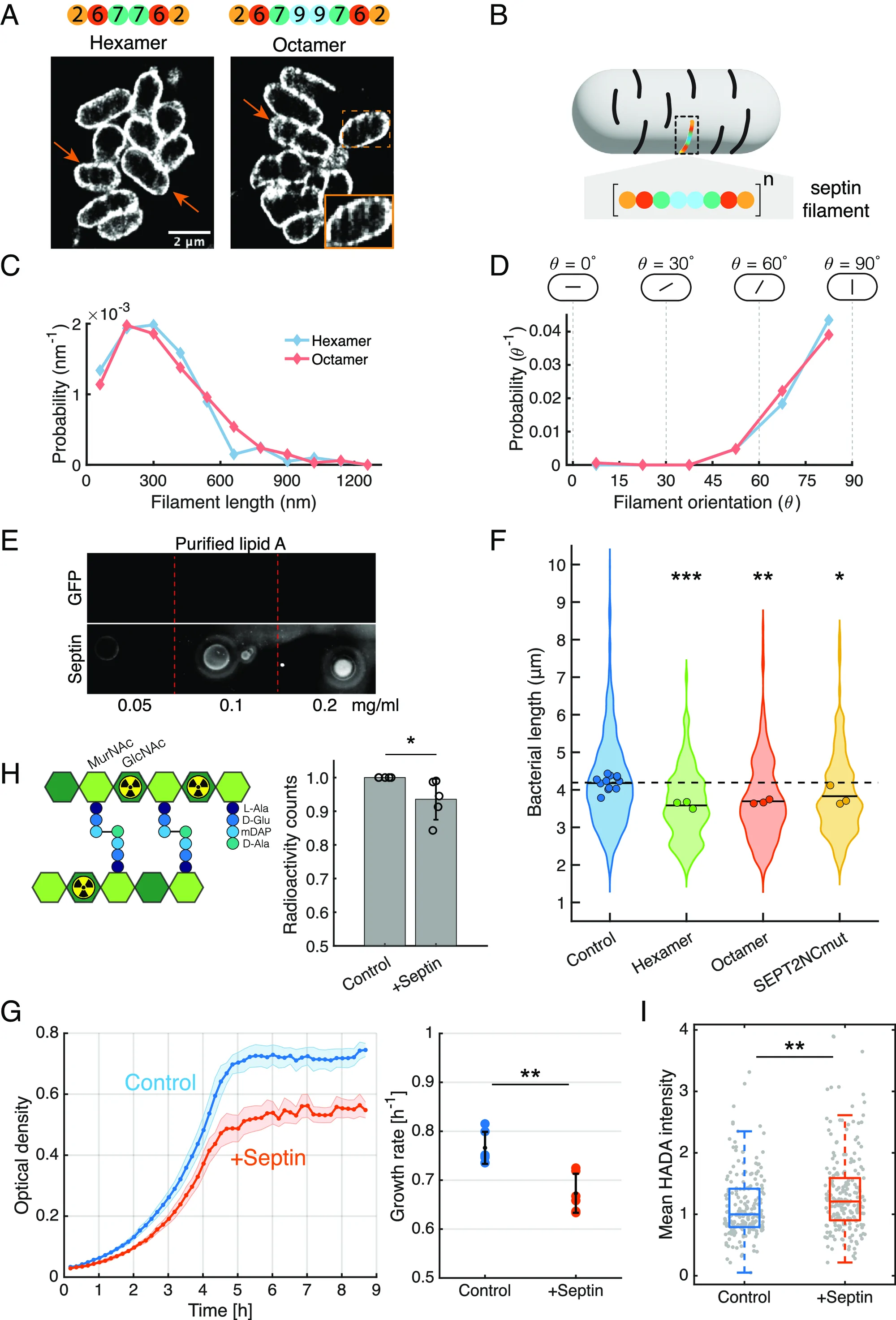
Septins assemble into filaments on the *Shigella* surface and inhibit bacterial growth. (*A*, *Top*) Illustration showing composition of septin hexamers (SEPT2-SEPT6-SEPT7-SEPT7-SEPT6-SEPT2) and septin octamers (SEPT2-SEPT6-SEPT7-SEPT9-SEPT9-SEPT7-SEPT6-SEPT2). (*Bottom*) SMLM reconstructed images show septins in a group of cells after in vitro septin-binding assays where several filaments per cell can be observed, indicated by orange arrows. Inset shows a zoomed-in image of a cell highlighted in dashed rectangle. (*B*) Cartoon demonstrating septin filaments arranged on the bacterial surface as short and disconnected filaments following near circumferential orientation. (*C*) Probability density distribution of filament lengths and (*D*) filament orientations. Four different orientations (0°, 30°, 60°, 90°) are depicted for visual guidance. (*E*) Two dot blots spotted with three different purified lipid A concentrations and incubated either with purified GFP or GFP-tagged octamer, displayed with the same image contrast. (*F*) Violin plots show probability distributions of cell length measurements which are collected from different biological replicates. Mean of each biological replicate is shown as darker shade circles with black outline. Minimum three biological replicates per condition. Pairwise comparisons to control are made by comparing means of biological replicates. *** shows *P* < 0.0005, ** shows *P* < 0.005 and * shows *P* < 0.05 according to one-way ANOVA with Dunnett’s multiple-comparisons test. (*G*, *Left*) Growth curves for control and septin-treated bacteria. Solid line shows mean and shaded area indicates SD between minimum three biological replicates. (*Right*) Growth rates estimated from exponential growth phases of each condition. ** shows *P* < 0.005 according to Student’s *t* test. (*H*, *Left*) Cartoon shows peptidoglycan cell wall structure in *Shigella* and highlights the cell wall component that is radioactively labeled. (*Right*) Average radioactivity levels observed in septin-treated condition normalized by the control population. Error bar represents mean ± SD of five biological replicates. * shows *P* < 0.05 according to one-tailed Student’s *t* test. (*I*) Mean HADA intensity normalized by median of control in control and septin-treated samples. Gray points represent single cells; box plots show median and data spread. ** shows *P* < 0.005 according to two-sided Welch’s *t* tests. *Shigella* ∆rfaC strain is used for these experiments.

We developed a cell-free in vitro reconstitution system to investigate how purified septin complexes are recruited to the surface of *Shigella* in the absence of other host factors (Fig. 1*A*) (15). Despite successful visualization of septin recruitment to *Shigella* by fluorescence microscopy and cryoelectron tomography (15), the spatial distribution of septin complexes was unclear. Using an LPS mutant ∆*rfaC* to maximize septin binding (15), we employed a custom-built single-molecule localization microscope to reveal that heteromeric septin complexes form higher-order assemblies on the bacterial surface (Fig. 1 *A* and *B*). We observed a broad distribution of filament lengths where averages measured as 320 nm and 339 nm for hexamer- and octamer-based complexes, respectively (Fig. 1*C*). Filaments were oriented near-perpendicular to the cell axis (78° and 75° for hexamers and octamers, respectively), indicating a strong preference for alignment around the cell circumference along the greatest principal curvature (Fig. 1*D*). Using an octamer complex containing mutant SEPT2 (SEPT2NCmut) that carries two point mutations disabling octamer–octamer interactions that results in lack of polymerization (20), we were no longer able to visualize filaments (*SI Appendix*, Fig. S1*A* and Movies S1 and S2). Together, these results highlight the ability of septins to assemble into filaments on bacterial membranes, as well as a universal property of septins as curvature sensors.

How septins interact with *Shigella* was mostly unknown. Studies using in vitro reconstitution of septin filaments on synthetic membranes showed higher affinity of septins to membranes containing anionic lipids, such as phosphatidylserine or phosphatidylinositol 4,5-bisphosphate (PI(4,5)P2), suggesting that septin–membrane interactions have electrostatic origins (24). Considering this, we hypothesized that septins bind negatively charged lipid A, a highly immunogenic bacterial molecule (25–27), on the outer membrane of *Shigella*. Since lipid A is embedded at the bottom of long lipopolysaccharide (LPS) chains (*SI Appendix*, Fig. S1*B*), shorter LPS structures would increase physical accessibility of lipid A and enable septin binding. To test this, we incubated wildtype (WT), ∆*rfaL*, ∆*galU,* and ∆*rfac* cells [i.e., cells with progressively shorter LPS chains (15)] with a lipid A antibody. Monitoring fluorescent signal showed that WT cells displayed practically zero lipid A staining while ∆*rfaL*, ∆*galU,* and ∆*rfac* mutants displayed progressively higher fluorescence intensities (*SI Appendix*, Fig. S1*C*). Next, upon incubation of mutants with septins, we observed levels of septin binding correlated with increased lipid A signal, supporting our hypothesis that septins interact with lipid A. To test this further, we applied a custom membrane lipid-strip method in which a nitrocellulose membrane is spotted with purified lipid A. Incubating the membrane with GFP-tagged purified septin complexes revealed high levels of GFP signal on lipid A containing droplets (Fig. 1*E*). As a control, incubation of the lipid-strip with purified GFP led to nearly 20-fold lower fluorescence intensity levels. From these data, we conclude that lipid A is likely one of multiple factors that can mediate septin binding and septins bind to lipid A when it is physically accessible.

### Septin Binding Inhibits Growth of *Shigella*.

Bacterial cell shape and growth dynamics are critical determinants of bacterial fitness and pathogenicity. We wondered if binding of septin filaments on bacteria would impact cell shape and growth, and analyzed the shape of bacteria that grew in the absence and presence of purified septin complexes. To ensure reliable measurements of bacterial growth, we blocked cell division using cephalexin, leaving cell elongation as the sole form of growth. Single-cell measurements after 2 h of incubation revealed significantly less elongation in the presence of septin binding, with average final lengths of 3.6 μm and 3.7 μm for hexamer- and octamer- entrapped bacteria, respectively, as compared to 4.2 μm in control bacteria (Fig. 1*F*). Despite changes in cell length, we found no changes in cell width (*SI Appendix*, Fig. S2*A*). Next, we quantified bacterial growth in the absence of cell division inhibitor using plate reader assays, a more conventional method for continuous growth monitoring. Results also showed that septin binding inhibits bacterial growth (Fig. 1 *G*, *Left*), reducing growth rate from 0.76 ± 0.03 in the absence of septin binding to 0.67 ± 0.03 in its presence (Fig. 1 *G*, *Right* and *SI Appendix*, Fig. S2*B*). As a control, we incubated bacteria with purified GFP alone and did not observe any reduction in growth rate (*SI Appendix*, Fig. S2*C*). Considering that mean fluorescence intensity at bacterial surfaces increased at higher septin concentrations (*SI Appendix*, Fig. S2 *D*, *Left*), we asked whether impact of septin binding is also increasing with concentration. However, bacteria incubated with either low or high amount of septins had similar mean cell lengths (*SI Appendix*, Fig. S2 *D*, *Right*), indicating that higher concentrations do not further restrict growth under these conditions.

Filament properties such as length, thickness, and orientation support a mechanical function for septins in inhibiting cell elongation. To test whether septin-mediated growth inhibition depends on filament formation, we measured bacterial elongation after incubating cells with mutant septins (SEPT2NCmut, no filaments). In this case, we observed partial growth inhibition with average final length of 3.9 μm (Fig. 1*F*), suggesting that hetero-oligomeric septin binding can restrict bacterial growth but filament formation is required for maximum impact.

The restriction of bacterial growth can result from inhibition of cell-envelope biosynthesis. To investigate the impact of septin binding on cell-envelope growth, we used radioactive labeling of the peptidoglycan cell wall with GlcNAc, a key component of peptidoglycan in Gram-negative bacteria (28, 29) (Fig. 1 *H*, *Left*). Exponentially growing cells were incubated with i) radioactive [^14^C]-GlcNAc alone or ii) both [^14^C]-GlcNAc and purified septin complexes. Following incubation, samples were processed to quantify incorporation of radioactive material into the peptidoglycan cell wall. We observed a 6.4% reduction of radioactivity incorporation in septin-bound *Shigella* compared to freely growing controls (Fig. 1 *H*, *Right*). Another key feature of bacterial cell growth is cell-envelope expansion. To determine whether cell-envelope expansion is affected by septin binding, we performed a labeling–dilution assay using HADA, a fluorescent D-amino acid that labels peptidoglycan. Bacteria were uniformly labeled with HADA in liquid culture, washed to remove unincorporated probe, and incubated in the absence or presence of purified septin complexes. As newly synthesized peptidoglycan remained unlabeled during the incubation period, cell-envelope expansion progressively diluted initial HADA signal. Consequently, cells undergoing greater expansion displayed lower fluorescence. Consistent with our previous observations, septin-bound *Shigella* retained 20% higher fluorescence than control cells, indicating reduced cell-envelope expansion (Fig. 1*I*). Together, these results show that septin-mediated growth restriction leads to reduced global cell-wall biosynthesis, establishing direct antibacterial activity of septin binding.

### Septin Binding to *Shigella* Upregulates T3SS Effectors and Triggers Stress Response.

**Fig. 2.**
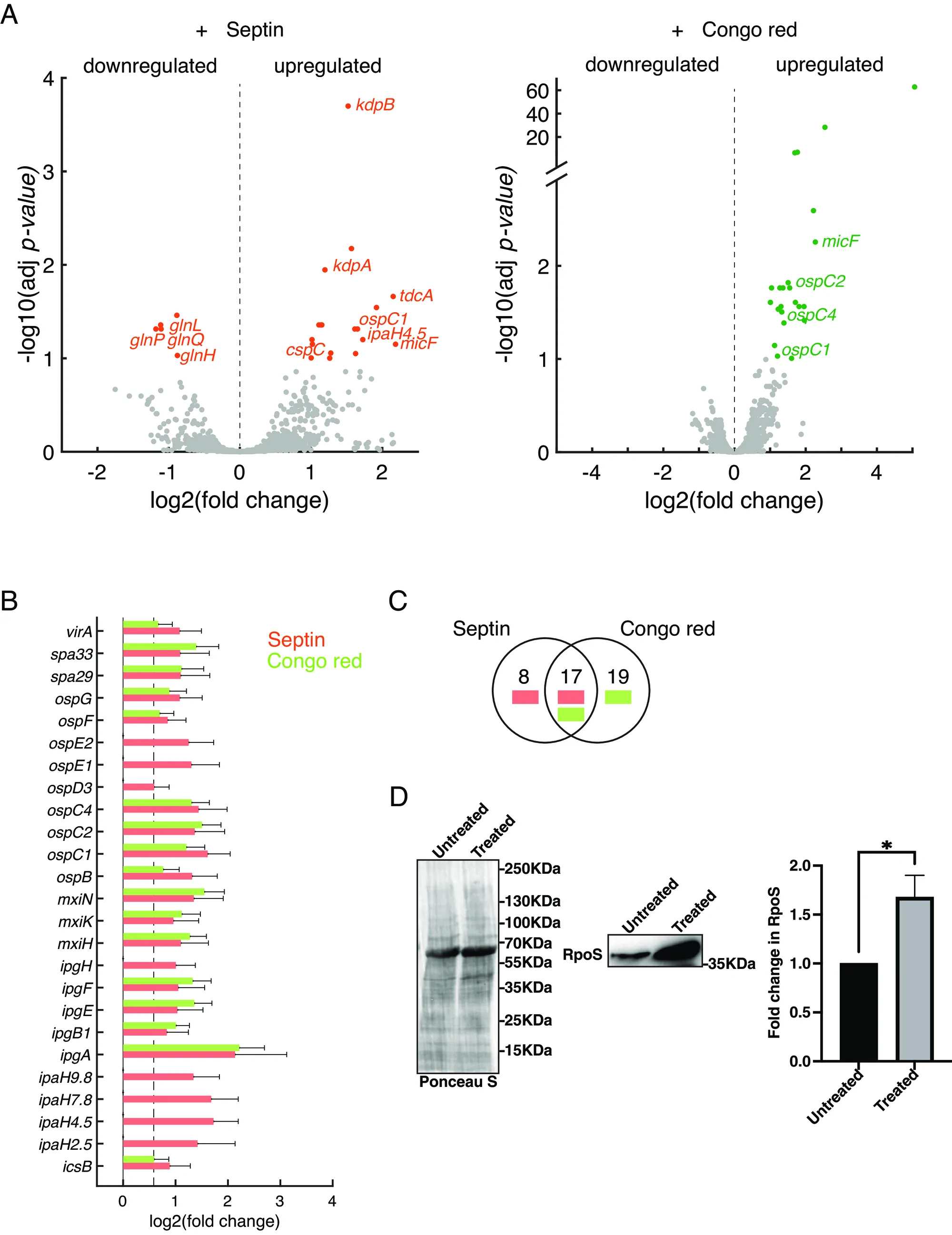
Transcriptional signature of *Shigella* upon septin binding. (*A*) Volcano plot (fold change between samples vs. adjusted *P*-value) showing differentially regulated genes in *Shigella* ∆rfaC upon (*Left*) septin binding and (*Right*) Congo red treatment in comparison to control samples. Statistically significant genes (*adj P*-value < 0.1 given by DEseq2) are shown as colored dots where gene names specified in the main text are labeled. (*B*) Bar plot shows the 25 T3SS genes which displayed fold change greater than 1.5 (a guide is given as dashed black line) and *P*-value < 0.05 given by DESeq2 upon septin treatment. Genes with transcriptional changes upon septin binding are shown in orange, fold change under Congo red treatment is shown in green when also significantly upregulated. (*C*) Venn diagram showing shared and unique T3SS genes upregulated by septin and Congo red treatment. (*D*) Ponceau S staining and western blot against RpoS are shown. RpoS band intensities are normalized by total protein counts from Ponceau S staining of corresponding condition. Fold changes are plotted. * shows *P* < 0.05 according to one-tailed Student’s *t* test.

We hypothesized that septin binding directly alters *Shigella* physiology, leading to reduced growth and cell-wall biosynthesis. To test this, we performed transcriptome sequencing (RNA-sequencing) of *Shigella* in the absence and presence of septin binding. Differential gene expression analysis revealed a dramatic shift in the global transcriptome of *Shigella* upon septin binding (Fig. 2 *A*, *Left* and *SI Appendix*, Table S2), and we confirmed changes in expression of key genes by qRT-PCR (*SI Appendix*, Fig. S3).

Unexpectedly, we found that septin binding upregulates a subset of T3SS effectors (Fig. 2 *A*, *Left*) where OspC1, shown to block interferon (IFN) signaling and expression of interferon-stimulated genes (ISGs) (30), was most significantly upregulated. Focusing on changes in expression levels of all known T3SS effectors revealed that 45 out of 57 total genes were upregulated >1.5-fold, including 25 with *P <* 0.05 (Fig. 2*B* and *SI Appendix*, Table S3). The upregulation of virulence genes indicates that bacteria sense septin binding as a host-related danger signal. To test this, we treated *Shigella* with Congo red, a historically used T3SS stimulant thought to function by mimicking a host tissue factor (31) and performed RNA-sequencing (Fig. 2 *A*, *Right*). As expected, examination of differentially expressed genes upon Congo red stimulation showed 39 genes upregulated by >1.5-fold, including 36 with *P <* 0.05 (Fig. 2*B* and *SI Appendix*, Table S4). Comparing RNA-sequencing profiles of bacteria upon septin- and Congo red-treatment showed a striking overlap with 17 T3SS effector genes (Fig. 2*C*), supporting our hypothesis that septins are sensed by bacteria as a host-related danger signal.

RNA-sequencing further revealed a stress response triggered by upregulation of *cspC*, involved in expression of sigma factor *rpoS* (global stress response regulator) (32), and upregulation of *tdcA,* associated with *cspC* expression and virulence of pathogenic bacteria (33, 34). Supporting this, experiments using western blot revealed significantly increased RpoS levels in septin-bound *Shigella*, indicating activation of the RpoS pathway (Fig. 2*D*). We found downregulation of *glnHLPQ* (glutamine transport operon), indicating changes to ion transport and cellular metabolism (35), and upregulation of *micF* (negative regulator of outer membrane porin OmpF), suggesting an adaptive response to environmental change (such as osmotic stress) to limit influx of harmful compounds. In addition, we found upregulation of *kdpA* and *kdpB*, part of a high affinity ATP-driven potassium transport system. Together, the transcriptomic response of *Shigella* points to mechanisms underlying growth inhibition and indicates that cells are under pressure due to septin binding, triggering a mechanism to tolerate cell-autonomous immunity.

### Septin Binding Affects Membrane Potential.

**Fig. 3.**
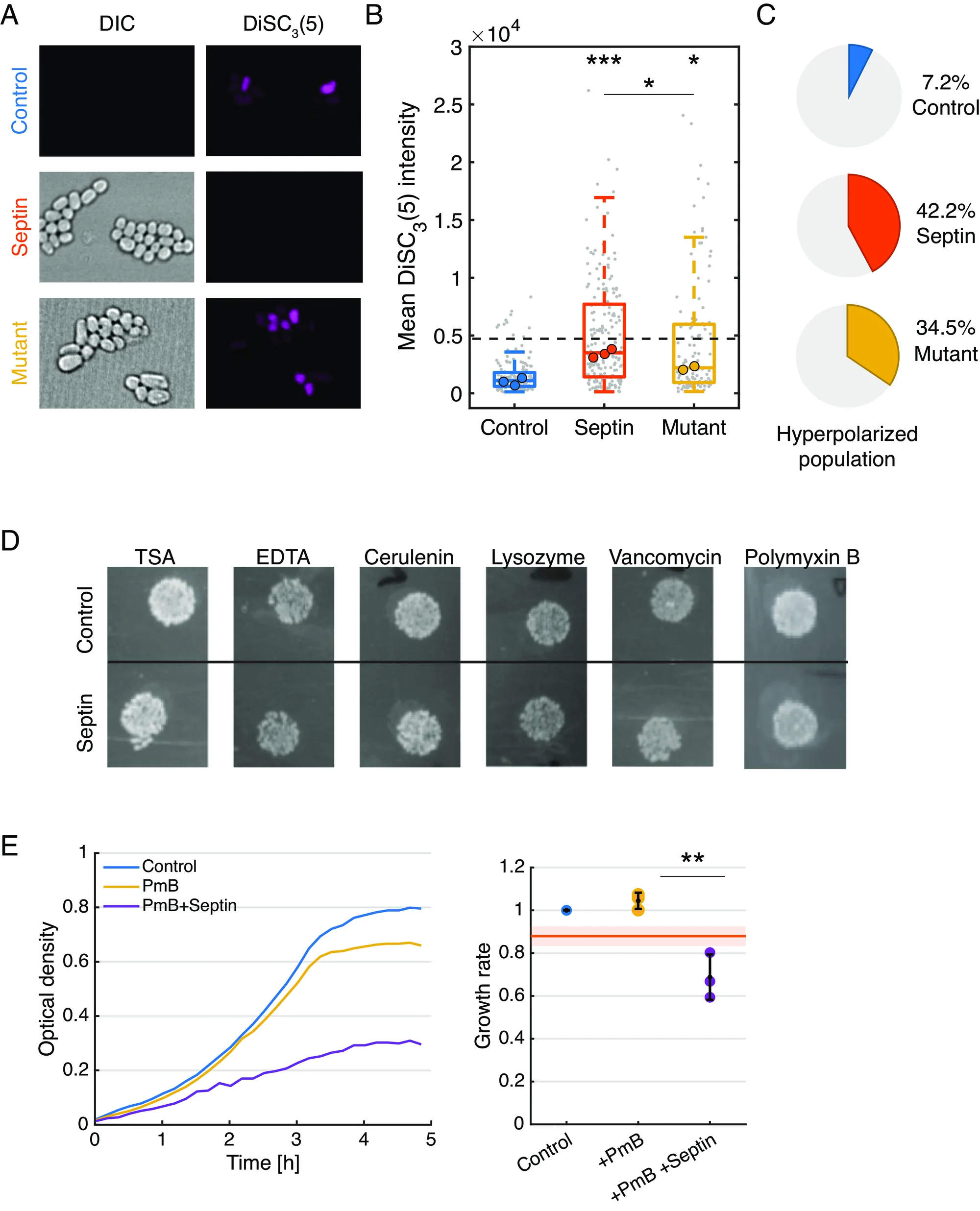
Septin binding promotes hyperpolarized membranes but not lethal cell envelope damage. (*A*) DIC (*Left*) and fluorescence microscopy (*Right*) images showing groups of *Shigella* ∆rfaC cells and corresponding DiSC_3_(5) staining. Fluorescence images are displayed with the same image contrast. Septin-bound bacteria exhibit highest intensity levels as quantified in (*B*). (*B*) Single-cell mean DiSC_3_(5) intensity values are plotted as gray circles and median of biological replicates are shown as colored larger circles with black outline. Hyperpolarization cutoff is calculated as two SD from the mean and plotted in a dashed black line. Pairwise comparisons are made by comparing means of biological replicates. *** shows *P* < 0.0005 and * shows *P* < 0.05 according to one-way ANOVA with Dunnett’s multiple-comparisons test. (*C*) Pie charts demonstrate percentages of hyperpolarized population observed in each condition. (*D*) Images of bacterial colonies preincubated with and without septins on TSA plates supplemented with no antibiotics, EDTA (0.25 mM, causes membrane destabilization), cerulenin (2 μg/mL, inhibits fatty acid synthesis), lysozyme (20 μg/mL, cleaves bonds in peptidoglycan), vancomycin (25 μg/mL, inhibits peptidoglycan synthesis of permeable Gram-negative bacteria), polymyxin B (0.05 μg/mL, destabilizes outer and cytoplasmic membranes). (*E*, *Left*) Growth curves for untreated control bacteria and bacteria treated with either polymyxin B, or septin plus polymyxin B. (*Right*) Growth rates were estimated from exponential phase of each growth condition and normalized to control for visualization. Each dot represents a biological replicate. The solid line and shaded area indicate mean and SD of growth rate of septin treated bacteria (as shown in Fig. 1*G*). ** shows *P* < 0.01 according to one-way ANOVA with Dunnett’s multiple-comparisons test. *Shigella* ∆rfaC strain is used for these experiments.

Bacterial membrane potential plays a key role in cellular metabolism. It is maintained by ionic gradients and regulated by ion channels, pumps, and cellular respiration, with potassium being essential to its maintenance. Considering the transcriptome of septin-bound *Shigella*, we tested the impact of septin binding on membrane potential using an established voltage-sensitive dye 3,3’-Dipropylthiadicarbocyanine Iodide (DiSC_3_(5)) which accumulates in polarized cells (Fig. 3*A*). Single-cell fluorescence microscopy measurements of DiSC_3_(5) signal revealed that average intensity is 3.4 times higher in bacteria bound by septins, as compared to control cells (Fig. 3*B*). Considering an intensity cutoff based on the control population (two SD from the mean), we found a hyperpolarized population of 7% in untreated cells as opposed to 42% in septin-treated cells (Fig. 3*C*). To confirm that DiSC_3_(5) reports membrane potential reliably, we treated freely growing *Shigella* with carbonyl cyanide m-chlorophenyl hydrazone (CCCP), a drug that rapidly dissipates membrane potential leading to depolarized cells, and observed a sharp decrease in DiSC_3_(5) signal, as expected (*SI Appendix*, Fig. S4*A*). In parallel we used thioflavin T, a fluorescent cationic dye that accumulates in cells due to negative electrical membrane potential. Similarly, we observed significantly increased thioflavin T signal for septin-treated cells, demonstrating a state of hyperpolarization (*SI Appendix*, Fig. S4*B*). To test a role for filament formation, we used mutant septins (SEPT2NCmut, no filaments) and observed a 2.2-fold increase in mean DiSC_3_(5) intensity, leading to a hyperpolarized population of 34% (Fig. 3 *B* and *C*). These results are consistent with growth inhibition results, showing that septin binding is partly able to alter membrane potential but filament formation is required for maximum antibacterial activity.

To determine whether these findings extend to other intracellular bacteria, we examined *Salmonella enterica* serovar Typhimurium (*Salmonella*), a widely accepted paradigm for studying cell-autonomous immunity. Using the same in vitro septin-binding assay, we observed septin recruitment to WT *Salmonella* and LPS mutant Δ*rfaJ* (*SI Appendix*, Fig. S5*A*). To establish relevance during infection, we infected HeLa cells and confirmed septin recruitment to both WT and LPS-mutant ∆*rfaJ* (*SI Appendix*, Fig. S5*B*). We selected Δ*rfaJ* for downstream analyses because its altered LPS structure increases outer-membrane accessibility, similar to *Shigella* Δ*rfaC* (*SI Appendix*, Fig. S5*C*). We tested whether septin binding similarly hyperpolarizes the *Salmonella* membrane. Measurements of DiSC_3_(5) uptake in the presence or absence of septins revealed a significantly higher membrane potential in septin-bound *Salmonella* (*SI Appendix*, Fig. S5*D*), as observed in *Shigella*, highlighting that membrane hyperpolarization is a general attribute of septin binding.

We next wondered if septin binding can also induce cell-envelope damage, leading to increased sensitivity to cell-envelope targeting antibiotics. To test this, we preincubated *Shigella* with either septins or control buffer and subsequently plated bacteria on tryptic soy agar (TSA) plates supplemented with various cell envelope targeting compounds (Ethylenediaminetetraacetic acid (EDTA), cerulenin, lysozyme, vancomycin), and interrogated cooperative effects by comparing sizes of cell growth spot (Fig. 3*D*). Preincubation with septins did not increase sensitivity to any of the molecules under the conditions tested, suggesting that septin-induced perturbations are reversible and do not cause lasting, lethal damage to cell envelopes under these conditions. To examine whether sustained exposure to septins increases sensitivity, we used plate-reader growth assays in which either subminimum inhibitory concentrations (sub-MICs) of polymyxin B (0.05 μg/mL) or both septins and polymyxin B were maintained in growth media. Strikingly, growth-inhibitory effects of polymyxin B were enhanced in the presence of septins (Fig. 3*E*), indicating that continuous septin binding sensitizes *Shigella* to cell-envelope-targeting antibiotics, presumably due to septin-induced changes in membrane potential.

### Intracellular *Shigella* Can Evade Septin Binding and Tolerate Cell-Autonomous Immunity.

**Fig. 4.**
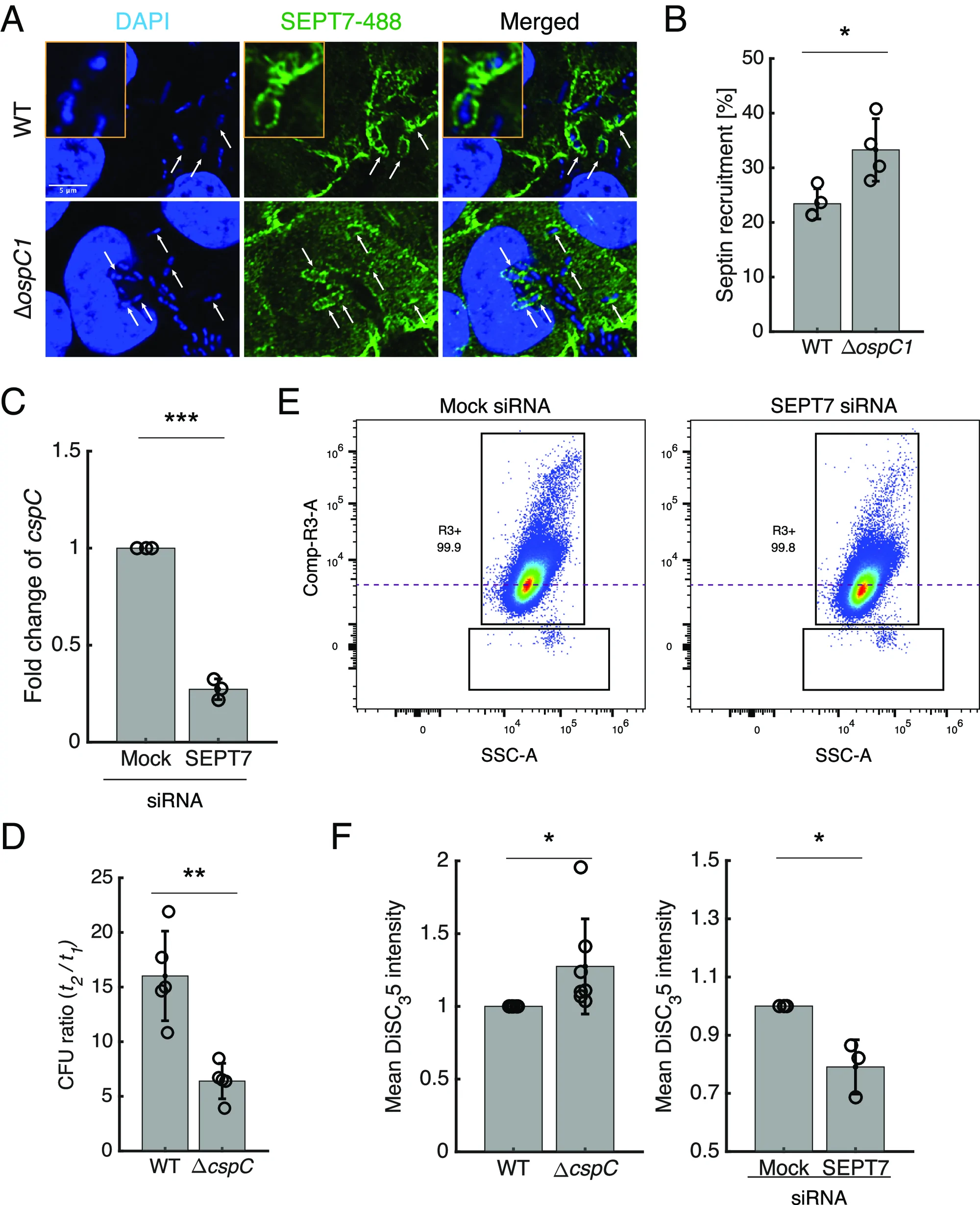
Intracellular *Shigella* can evade septin binding and tolerate cell-autonomous immunity. (*A*) Representative images of septin-bound bacteria in HeLa cells infected with either WT or Δ*ospC1 Shigella*, 3 h 40 min postinfection. White arrows indicate septin-bound bacteria. Insets show a zoomed-in image of two septin-caged WT bacteria. (*B*) ∆*ospC1* mutant exhibit higher levels of septin binding during *Shigella* infection as shown by septin-positive percentage of intracellular *Shigella*. *N* = 761, 1,087, 825, 760 and *N* = 1,302, 765, 502, 524 for WT and mutant, respectively. Each biological replicate is shown in black circles. Error bars represent mean ± SD of four biological replicates. (*C*) Fold change of *cspC* expression of intracellular *Shigella* (∆*ospC1*, to maximize septin binding) after HeLa infection in control and SEPT7 depleted cells. *cspC* is upregulated in intracellular bacteria where SEPT7 is not depleted. (*D*) ∆*cspC* (*cspC::kanR*) mutant replicates poorly during infection as shown by the comparison of CFU ratios (*t*_2_ for 3 h 40 min and *t*_1_ for 1 h 40 min) of *Shigella* during HeLa infection. Black circles represent biological replicates and error bars show mean ± SD. (*E*) Representative scatter plots of mean fluorescence intensities (MFI, *y*-axis) of DiSC_3_(5) stained intracellular *Shigella* (∆*cspC*) observed by flow cytometry. Bacterial cells are collected from infected HeLa cells treated with either mock siRNA (*Left*) or SEPT7 siRNA (*Right*). A dashed line is drawn as a visual guide to help comparison of scatter plots. *** shows *P* < 0.0005, ** shows *P* < 0.005 * shows *P* < 0.05 according to Student’s *t* test. (*F*) Mean MFI values per biological replicate of DiSC_3_(5) stained intracellular *Shigella*. To account for day-to-day variation in absolute fluorescence intensity, each dataset is normalized to the corresponding control condition collected on the same day. (*Left*) Comparing averages obtained from WT and ∆*cspC* shows increased fluorescence intensity for the mutant. (*Right*) ∆*cspC Shigella* displays higher membrane polarization in HeLa cells where SEPT7 is not depleted. Black circles represent biological replicates. *N* > 25,000 single bacterial cells. Error bars show mean ± SD of biological replicates.

The changes observed upon septin binding in vitro suggest that a bacterial mechanism for tolerating cell-autonomous immunity is triggered. To study the impact of our findings during infection, we turned to *Shigella* infection of human epithelial cells. We first tested the role of OspC1 in *Shigella*–septin interactions. We compared the percentages of septin-bound bacteria in cells infected with either WT or ∆*ospC1 Shigella* and revealed significantly higher septin recruitment in ∆*ospC1* infected cells (22% and 33%, respectively), supporting a role for OspC1 as a septin-targeting virulence effector (Fig. 4 *A* and *B*). Together with recent work showing that OspC effectors catalyze ADP-riboxination of SEPT9 (36), these findings highlight a requirement for octameric complexes in septin-mediated cell-autonomous immunity and its evasion by OspC1.

To validate our in vitro findings in cells, we performed qRT-PCR on intracellular bacteria to quantify expression of stress response gene *cspC* following siRNA-mediated depletion of either a nontargeting control (mock) or SEPT7 (essential component of both hexameric and octameric septin complexes). To maximize the proportion of bacteria associated with septins, we exploited our finding that OspC1 inhibits septin cage entrapment and used the ∆*ospC1 Shigella* strain we developed. Under these conditions, *cspC* expression was 3.8-fold higher in the presence of SEPT7 than following SEPT7 depletion (Fig. 4*C*), supporting our in vitro observations that septin binding to bacterial membrane triggers a stress response.

We finally investigated if *cspC*-regulated stress response is crucial for intracellular replication during infection. Monitoring bacterial replication rates during infection revealed that WT bacteria led to 2.5-fold higher bacterial load than a ∆*cspC* mutant (*cspC::kanR*) (Fig. 4*D*). To examine whether the membrane potential of ∆*cspC* mutant is maintained as WT during infection, we measured DiSC_3_(5) fluorescence levels of single intracellular bacteria using flow cytometry (Fig. 4*E*). Strikingly, we observed elevated DiSC_3_(5) levels for *cspC* mutant (Fig. 4 *F*, *Left*). As a control, we compared growth rates and DiSC_3_(5) uptake of WT and Δ*cspC* bacteria and observed no significant differences (*SI Appendix*, Fig. S6 *A* and *B*). We also confirmed that bacterial load did not affect DiSC_3_(5) uptake by infecting HeLa cells at multiplicities of infection (MOIs) of 10 and 100 (*SI Appendix*, Fig. S6*C*). To investigate a role for septin binding in differential regulation of membrane potential in situ, we measured DiSC_3_(5) signal in SEPT7-depleted cells using silencing RNA. In agreement with our findings in vitro, we observed significantly higher levels of DiSC_3_(5) fluorescence intensity in the presence of septin expression (Fig. 4 *F*, *Right*). Given reduced capacity of *cspC* mutant for intracellular replication, we conclude that septin-mediated changes in *Shigella* physiology is a tolerance mechanism for bacteria to survive cell-autonomous immunity.

## Discussion

In this work, we find that septin binding and filament formation has direct antibacterial activity by inhibiting bacterial growth. Septin-bound *Shigella* exhibited transcriptomic changes such as activation of stress response pathways and increased T3SS expression, supported by independent findings of reduced cell length, decreased peptidoglycan incorporation, and increased membrane potential. Together, these linked phenotypes support a model in which septin binding imposes a significant physiological stress on *Shigella*. Our data indicate that growth inhibition arises from septin-induced stress response. Mechanistic uncoupling of septin binding from filament formation further indicates that binding itself can partially inhibit growth, whereas filament formation is needed for maximum impact, potentially by contributing to binding stability on the bacterial surface.

We show that T3SS effector *ospC* helps to evade septin cage entrapment, and *cspC* stress response is required for survival from septin-mediated alterations to membrane potential. Importantly, bacterial response to septins reveals tolerance mechanisms otherwise impossible to observe with other cell-autonomous immune mechanisms such as GBPs where cell envelope loses its integrity (promoting rapid cell lysis upon binding) (8, 16). We also introduce the concept of cytoplasmic bacterial tolerance to cell-autonomous immunity as essential to consider for infection control.

Our findings reveal that septin binding to bacteria controls membrane potential. In situ, this interaction may modulate ion fluxes during infection by altering bacterial membrane potential, thereby leaving cytoplasmic bacteria more susceptible to antimicrobial peptides. By using bacterial plating assays, we show that physiological impact of septin binding on *Shigella* is reversible while continuous exposure to septins make them more susceptible to polymyxin B, a cationic antimicrobial peptide, potentially due to membrane hyperpolarization. Future work will test a wide range of antibiotics in the absence and presence of septin binding to assess mechanisms underlying increased antibiotic sensitivity. It is also of particular interest to examine parallels for septin roles in eukaryotic cells, where septin–membrane interactions are widely observed (18). As one example, understanding how septin–membrane interactions modulate neuronal ion fluxes and electrical signaling could provide fundamental mechanistic insights underlying septin-mediated neuropathies in humans.

Environment sensing is key for bacteria and their survival as they mostly reside in external environments. The ability of sensing danger enables bacteria to adapt and survive stressful conditions. For example, it was shown that RpoS-mediated stress response helps cells during hostile conditions and also prepares them for subsequent environmental threats, a concept known as bet-hedging (32). Recent work has significantly advanced our understanding of bacterial defense mechanisms against phage attacks (37, 38) but how pathogenic bacteria sense or recognize host cell attacks or components of cell-autonomous immunity is still largely unknown. Here we found that *Shigella* recognizes septin binding as a host-related danger signal and activates a stress response which enables bacterial survival. Is there a dedicated pathway in bacteria to sense host-related danger which proactively enables tolerance to harsh conditions of the cytoplasm? *Shigella* has been shown to significantly modify its lipid A structure during infection and may recognize septin binding to lipid A as a host-related danger signal. Supporting this, we previously observed a twofold increase in septin cage entrapment for ∆*rfaC* over WT bacteria during infection of HeLa cells (15), suggesting increased septin binding to *Shigella* where lipid A is more accessible. Septin-lipid A interactions may serve as a cue for both host and bacterial cells to activate survival mechanisms. Unfortunately, tools to study lipid A in situ are limited due to complexity of bacterial biosynthetic pathways that introduce chemical modifications to lipid A and LPS structures (39). It will next be of great interest to investigate intracellular tolerance in other important human pathogens that replicate in the cytoplasm (such as *Salmonella* Typhimurium and *Listeria* *monocytogenes*), and to other mechanisms of cell-autonomous immunity (such as antibacterial autophagy and RNF213-mediated ubiquitination). In agreement, we show that septin binding to *Salmonella* results in membrane hyperpolarization. These insights may inform strategies to combat *Shigella* and other WHO high-priority pathogens.

## Materials and Methods

### Reagents.

Antibodies used in this work include anti-human Septin 7 rabbit IgG (Tecan, IBL, JP18991), anti-*Escherichia coli* J5 LPS antibody (Tebubio, DS-MB-01267), FluoTag®-X4 anti-GFP (NanoTag Biotechnologies, N0304), purified anti-*E. coli* RNA sigma S antibody (BioLegend, 663706), and DnaK antibody (Enzo, ADI-SPA-880-D). Secondary antibodies include goat anti-rabbit IgG Alexa Fluor 488 (Thermo Fisher Scientific, A-11034), goat anti-mouse IgG Alexa Fluor 647 (Thermo Fisher Scientific, A-21236), and polyclonal goat anti-mouse immunoglobulins (HRP), (Agilent, P0447).

To deplete septins in HeLa cells we used oligofectamine (Invitrogen, 12252-011) and SEPT7 Silencer Predesigned siRNA (Thermo Fisher Scientific, 10289574 ID: 10323); Silencer siRNA negative control (Thermo Fisher Scientific, 10793457) was used as control.

Dyes used in this work include Congo red (Sigma-Aldrich, C6767), Hoechst (Thermo Fisher Scientific, H3570), 3,3′-Dipropylthiadicarbocyanine iodide (DiSC3(5), Merck, 43608), Thioflavin T (ThT, Thermo Fisher Scientific, 211760250), and fluorescent D-amino acid HADA (Tocris Bioscience, 6647).

We used purified lipid A (from *Salmonella enterica* serotype minnesota Re 595, Merck, L6895) for our dot blot assay. Carbonyl Cyanide m-Chlorophenylhydrazone (CCCP, Merck, C2759), fatty acid free bovine serum albumin (BSA) (Sigma-Aldrich, A6003), D-GlcNAc (Sigma-Aldrich, A8625) and *N*-acetyl-D-glucosamine [1-14C] ([^14^C]-GlcNAc, ARC, 0105-50 µCi) were used in this work.

### Bacterial Growth and Genetic Constructions.

*Shigella* *flexneri* serovar 5a strain M90T and its mutants were grown from −80 °C stocks on tryptic soy broth (TSB)-agar containing 0.01% (w/v) Congo red to select for red colonies, indicative of a functional T3SS, overnight at 37 °C. Corning 50 mL tubes containing 5 mL of growth medium were inoculated with individual red colonies of *Shigella* and were grown overnight at 37 °C with shaking at 200 rpm. The following day, bacterial cultures were diluted in fresh medium (1:200 v/v) and cultured until an OD_600_ between 0.3 and 0.4. For all in vitro reconstitution experiments, *Shigella* was grown in defined minimal medium containing [50 mM Tris-HCl pH 8.0, 50 mM KCl, 0.5 mM MgCl_2_, 0.1 mM CaCl_2_, 1 mM MgSO_4_, 45 μg/mL L-methionine, 20 μg/mL L-tryptophan, 12.5 μg/mL nicotinic acid, 10 μg/mL vitamin B1, 1% glucose, 0.5% casein hydrolysate, 0.1% fatty acid-free BSA]. For all human epithelial tissue culture infections, *Shigella* were grown in TSB.

*Salmonella enterica* serovar Typhimurium (NCTC 12023) and its mutants were grown from −80 °C stocks on LB-agar plates to select for single colonies. Corning 50 mL tubes containing 5 mL of growth medium were inoculated with individual colonies of *Salmonella* and were grown overnight at 37 °C. The following day, bacterial cultures were diluted in fresh medium (1:100 v/v) and cultured until an OD_600_ between 0.5 and 0.6. For all in vitro reconstitution experiments, *Salmonella* was grown in defined minimal medium (as above) during exposure to septins. For all human epithelial tissue culture infections, *Salmonella* were grown in LB.

The bacterial strains and plasmids used in this study are listed in *SI Appendix*, Table S1. Deletion mutants were constructed as previously described using pKD46 and pKD4 plasmids (15). Kanamycin-resistant colonies were picked and investigated by colony PCR.

The plasmid expressing SEPT2NCmut septin octamer (pmsGFP-SEPT6-HIS-SEPT2NCmut) was constructed by Gibson assembly (NEB, E5510S). Briefly, SEPT2NCmut was PCR-amplified from pCMV_SEPT2-NCmut-mApple using the primers 48_SEPT2NCmut_fwd and 49_SEPT2NCmut_rev. Plasmid backbone was PCR-amplified from pmsGFP-SEPT6-HIS-SEPT2 using the primers 46_pmsGFP-SEPT6_SEPT2_fwd and 47_pmsGFP-SEPT6_SEPT2_rev. After Gibson assembly, three plasmids were randomly selected and sequenced using the primers 50_SEPT2 and 72_SEPT2. The clone with the correct sequence was then used for septin purification.

### Septin Protein Purification.

Purification of GFP and septin hexamers was performed as described in ref. 15 and septin octamers as described in ref. 20. Purification results in preformed covalent heterooligomers such as hexamer, octamer, or SEPT2NCmut octamer as described in refs. 15 and 20. The storage buffer contains 50 mM Tris-HCl pH 8.0, 300 mM KCl, 5 mM MgCl_2_, and 1 mM DTT. We diluted purified septins 1:3 v/v in Tris buffer (50 mM, pH 8.0) right before mixing with bacteria for in vitro reconstitution to reduce the amount of salts. For control conditions, the same dilution was applied to the storage buffer.

### In Vitro Reconstitution Assay.

*Shigella* (M90T∆*rfaC*, unless otherwise stated) cultures growing in minimal medium were harvested at OD_600_ between 0.3 to 0.4 with 1.2 absorbance unit of bacteria (e.g., 3 mL of culture at OD_600_ 0.40). Cells were centrifuged in low protein binding tubes at 9,000 rpm for 1 min at room temperature and resuspended in fresh minimal medium. Next, purified septin complexes (240 nM) and DTT (1 mM) were added. The final volume of the reaction was set as 100 μL. As a control, we treated cells in the same way but replaced septin-containing solution with diluted storage buffer. Controls and septin-including samples were incubated at 37 °C for 2 h with shaking at 200 rpm. Next, samples were centrifuged and washed with fresh minimal medium twice to remove unbound septins. Controls were treated in the same way. For septin binding assays with *Salmonella*, we performed the same protocol, except that incubation was carried out under static conditions (40).

### Sample Preparation and Imaging with Single Molecule Localization Microscopy (SMLM).

After incubating *Shigella* with GFP-tagged septins as described in *In Vitro Reconstitution Assay*, cells were fixed with 4% PFA in PBS for 15 min at room temperature and washed three times with PBS. Next, samples were incubated with FluoTag®-X4 anti-GFP (NanoTag Biotechnologies, N0304) for 60 min at room temperature and washed three times with PBS buffer. The PFA-fixed sample was spotted onto a poly-lysine coated #1.5 coverslip (VWR, 6310172) for 30 min for cell attachment. The coverslip was then loaded onto a custom-made holder and immersed in 1× SMLM buffer, which contains 200 mM sodium sulfite, 10 µM mercaptoethylamine (MEA), and 100 mM β-mercaptoethanol (BME) in PBS (41).

A custom built SMLM microscope equipped with 60×/1.40 oil immersive Plan Apo objective was used (42). Specifically, a wide-field (WF) image was captured (200 ms exposure, 20 mW of 642 nm laser) first, followed by the SMLM imaging acquisition where 25,000 frames were taken (each with 10 ms exposure, 1,200 mW of 642 nm laser illumination). An astigmatism lens was used during imaging to separate individual fluorophores into 3-dimension (43). The 405 nm activation laser power that permits prolonged imaging was manually incremented to keep blinking frequencies constant.

Reconstruction of raw SMLM image stacks was processed with the ThunderSTORM plugin in Fiji (44, 45). The multiemitter fitting analysis was enabled with a maximum of three molecules per fitting region. Drift correction was achieved by the cross-correlation tool. Duplicates that converged to the same localization uncertainty were removed. Images were visualized by a sum of reconstructed slices using averaged shifted histograms with a pixel size of 40 nm.

### Filament Length and Orientation Measurements.

We used custom-written MATLAB scripts to semiautomatically analyze filament length and orientation. Single-molecule localizations obtained from ThunderSTORM were used to generate a 3D-reconstructed image. The images were visualized as 3D z-stacks, and regions resembling filaments were manually labeled by drawing straight lines on 2D images displaying all localizations. Signal intensity, given by the number of localizations, along these lines was then subjected to automated thresholding, with the cutoff defined by the median signal. Regions where signal consistently exceeded this threshold were classified as filaments. Filament length was calculated based on the spatial continuity of these high-signal regions. Orientation of each filament was found by computing the angle (*θ*) between each filament and the centerline (major axis) of the cell. All *θ* values were mapped between 0° and 90° since neither filaments nor bacterial cells exhibit directionality.

### Lipid A Staining and Dot Blot for Lipid A Binding.

In experiments where we investigated correlations between lipid A staining and septin binding, we first performed in vitro septin binding assay (as described in *In Vitro Reconstitution Assay*) followed by incubation with lipid A antibody (1:200 v/v of 0.5 mg/mL stock solution), followed by incubation with Alexa Fluor™ 647 (1:500 v/v of 2 mg/mL stock solution), each for 20 min at room temperature. We used 50 mM Tris pH 8.0 supplemented with 1% fatty-acid free BSA for diluting primary and secondary antibodies. Next, cells were washed twice by centrifugation, resuspended in fresh medium, and spotted on agarose pads for imaging.

A nitrocellulose membrane (BioRad, 1620145) was spotted with varying concentrations of purified lipid A (200 to 25 pmol) and a negative control of Tris buffer (50 mM, pH 8.0), then air-dried. It was blocked with 3% fatty acid–free BSA in Tris for 30 min at room temperature with gentle shaking, followed by three 5-min washes in Tris. The membrane was then incubated with 240 nM GFP or septin–GFP for 2 h under the same conditions and washed again three times (5 min each) to remove unbound proteins. GFP signals were detected using the BioRad ChemiDoc MP imaging system.

### Experimental Design and Bacterial Cell Shape Measurements.

The experimental design followed the approach described in *In Vitro Reconstitution Assay*, with the modification that the cell division inhibitor cefalexin (10 μg/mL) was added concurrently with either septins (treatment) or buffer (control). All samples were incubated for 2 h under the same conditions before harvesting for microscopy.

We used LSM 880 confocal (ZEISS) to acquire Differential Interference Contrast (DIC) microscopy images. Bacterial cell boundaries were detected by using the MATLAB based cell segmentation tool Morphometrics (SimTK) (46). A cell-internal coordinate system was identified using the MATLAB based MicrobeTracker package (47). Cell length was measured as the total length along the cell skeleton and cell width was measured as the median of all local widths using custom MATLAB scripts.

### Sample Preparation and Imaging with Airyscan Microscope.

Imaging was performed on LSM 880 laser scanning confocal microscope (ZEISS) equipped with a 63× Plan Apochromatic 1.4 NA oil objective, an Airyscan superresolution module, and driven by Zen Black acquisition software (ZEISS v2.3). Pixel size was 41 nm. Confocal images were processed using Airyscan processing (Weinerfilter) using “Auto Filter” option with Zen Black software.

For *In Vitro Reconstitution Assays*, live bacteria harvested for imaging were spotted on agarose pads (minimal medium supplemented with 1% agarose). Coverslips were sealed with vasoline: lanoline:paraffin (VALAP; 1:1:1) to prevent drying of the pads. The imaging time did not exceed 20 min per replicate.

### Plate Reader Growth Assays and Growth Rate Estimation.

Single colonies of *Shigella* (∆*rfaC*) were cultured overnight in minimal medium at 37 °C with shaking at 200 rpm. The following day, cultures were diluted 1:100 (v/v) in fresh medium and incubated in 50-mL Corning tubes for 2 h under the same conditions. Cultures were then transferred to 96-well plates at a final volume of 100 µL per well. Plates were incubated for 24 h at 37 °C with continuous shaking, and optical density (OD) was measured every 10 min using a BioTek Synergy 2 plate reader with Gen5 software. Final concentrations of septins and antibiotics are provided in corresponding figure legends.

Background-corrected OD values were plotted in figures and used to calculate growth rate. For each well, exponential growth phase was identified, and natural logarithm of OD was plotted as function of time. Specific growth rate (μ) was determined as slope of a linear regression fitted to this region, and doubling time was calculated as ln(2)/μ. Growth rates from biological replicates were then averaged for each experimental condition.

### Radiolabeling of Peptidoglycan with [^14^C]-GlcNAc.

Single colonies of *Shigella* (∆*rfaC*) were grown overnight in minimal medium at 37 °C with shaking at 200 rpm. The following day, bacterial cultures were diluted in fresh medium (1:200 v/v) supplemented with 0.2 mM GlcNAc and cultured until an OD_600_ between 0.3 and 0.4. Cells were then harvested for the septin-binding as described in *In Vitro Reconstitution Assay*. A control sample was always included where cells were incubated with the storage buffer. For [^14^C]-GlcNAc labeling of bacteria, both control and septin-added samples were spiked with 0.5 μCi/mL of *N*-acetyl-D-glucosamine [1-14C] ([^14^C]-GlcNAc; ARC 0105-50 µCi) and incubated for 2 h at 37 °C with shaking.

Processing of samples for radioactivity measurements was adapted from ref. 29. Briefly, samples were taken and boiled in 4% SDS for at least 1 h. After cooling the SDS samples to room temperature, they were filtered through 0.22 μm membrane filters (Millipore GSWP02500). The filters were then washed with 30 mL distilled water three times, collected into scintillation count vials and treated with 400 μL of 10 mg/mL lysozyme at 37 °C for at least 2 h, and finally dissolved in 5 mL scintillation cocktail (Filter‐Count, Perkin Elmers) overnight. Counts per minute due to radioactive decay of ^14^C were measured in a scintillation counter (TriCarb, Perkin Elmer). The radioactivity in the septin-treated sample was calculated by dividing its total counts by those of the control sample.

### Fluorescent Labeling of Peptidoglycan with HADA.

Exponentially growing *Shigella* were incubated with HADA (200 µM) for 1 h at 37 °C with shaking. Bacteria were then washed twice with prewarmed medium to remove excess HADA and harvested for the in vitro septin-binding assay.

### RNA-Sequencing Experimental Conditions and Data Analysis.

Previous work using siRNA showed that *Shigella*-septin cages were significantly reduced upon SEPT9 depletion in HeLa cells (14), identifying an important role for SEPT9 (hence octamers) in septin cage formation. Therefore, we decided to conduct RNA-sequencing experiments with octamer-based septin complexes which contain SEPT9.

The RNA-sequencing experimental conditions for the “Control” and “+Septin” samples were identical to those used in our microscopy experiments (described in *Experimental Design and Bacterial Cell Shape Measurements*), which demonstrated bacterial growth restriction. Septin-treated samples were verified for septin binding with fluorescence microscopy. For samples treated with Congo red, septins were replaced with Congo red (0.01%), while all other experimental parameters remained unchanged. RNA was extracted from snap-frozen bacterial samples (three biological replicates for each condition) with the Monarch Total RNA Miniprep Kit (NEB, T2010S) according to the manufacturer’s instructions. Sequencing was performed with Illumina NextSeq 2000 sequencing systems with paired-end reads.

Briefly, fastq files from RNA-sequencing were analyzed as follows: We used FastQC and Trimmomatic (48) tools for quality control and for trimming low-quality reads, respectively. Next, BWA-MEM (49) was used for aligning paired-end reads against the reference genome (GCA.000006925.2) and HTSeq-count (50) was used for calculating read coverage (counts) to genomic features. These counts were then analyzed with DEseq2 (51) for differential expression analysis. All reported fold changes, *P*-values and adjusted *P*-values are raw data provided by DEseq2 analysis. All data analysis was performed using the Galaxy Webserver (https://usegalaxy.eu/).

### qRT-PCR Sample Preparation and Data Analysis.

Using the same samples submitted to RNA-sequencing, RNA extraction was followed by reverse-transcription using QuantiTect Reverse Transcription kit (Qiagen, 205311) according to manufacturer’s instructions. qRT-PCR was performed using the QuantStudio 7 Pro (Thermo Fisher Scientific), Design & Analysis Software v2.8.0 (Thermo Fisher Scientific) and SYBR green master mix (Thermo Fisher Scientific, 4309155). Template cDNA was subjected to PCR using primers described in *SI Appendix*, Table S1, and samples were run in technical duplicates. Quantification was obtained using the 2*^−ΔΔ^*^CT^ method and *gyrB* was used as the reference gene.

To measure *cspC* expression during HeLa cell infection, cells were washed three times with PBS at 3 h 40 min postinfection and lysed with 0.1% Triton X-100 in PBS to release intracellular bacteria. Recovered bacteria were collected in Eppendorf tubes and washed with PBS. Resulting pellets were immediately frozen on dry ice for subsequent RNA extraction with Monarch Total RNA Miniprep Kit (NEB, T2010S) according to manufacturer’s instructions. Downstream processing is performed as above.

### Measurement of Membrane Potential.

For quantifying membrane potential of septin-treated *Shigella*, control and septin-treated samples were further incubated with DiSC_3_(5) (final concentration of 1 μM) after in vitro reconstitution assay, for 5 min at 37 °C with shaking. For ThT labeling, control and septin-treated samples were incubated with ThT (10 μM) after in vitro reconstitution assay, for 30 min at 37 °C with shaking. For CCCP treatment, we treated exponentially growing bacteria with 10 μg/mL of CCCP for 5 min and subsequently labeled with DiSC_3_(5) (final concentration of 1 μM) for 5 min at 37 °C with shaking. For all labeling experiments, bacteria were washed twice in fresh minimal medium and harvested for microscopy.

To measure the single-cell DiSC_3_(5) fluorescence signal of *Shigella*, we used Morphometrics to automatically detect bacterial cell boundaries. Cells with adjacent neighbors were eliminated to ensure accurate measurements of single-cell fluorescence. The detected cell boundaries were converted into masks using the built-in poly2mask function in MATLAB. The fluorescent images were filtered with a 2D-Gaussian filter of size 11 and SD of 0.5 pixels. Pixel values within the single-cell masks were then extracted and averaged to obtain mean fluorescence values.

For bacterial membrane potential measurements during infection, HeLa cells were infected with *Shigella* strains as described in *Human Cell Tissue Culture and Infections*. DiSC_3_(5) was added to HeLa cells seeded in 6-well plates 30 min prior to PFA fixation at a final concentration of 1 μM. At 3 h 40 min post infection, cells were fixed and washed three times with PBS, and 0.1% Triton X-100 (in PBS) was used for cell lysis and release of intracellular bacteria. Fixed bacteria were collected in Eppendorf tubes and harvested for flow cytometry.

### Antibiotic Sensitivity Test for Cell Envelope Damage.

For TSB-agar plate based assays, control and septin-treated samples after in vitro reconstitution assay were subjected to serial dilutions in fresh medium. Next, 5 μL of diluted samples were spotted on TSB-agar plates that contained either no antibiotics, EDTA (0.25 mM), lysozyme (20 μg/mL), vancomycin (25 μg/mL), or cerulenin (2 μg/mL). Final concentrations of each chemical are given in parenthesis. Plates were incubated at 37 °C overnight and CFU counts were compared on the next day.

For plate reader based assays, plates are prepared as described in *Plate Reader Growth Assays and Growth Rate Estimation*.

### Human Cell Tissue Culture and Infections.

HeLa (ATCC CCL-2) cells were grown at 37 °C and 5% CO_2_ in Dulbecco’s modified Eagle medium (DMEM, GIBCO) supplemented with 10% fetal bovine serum (FBS, Sigma-Aldrich).

*Shigella* infections were adapted from ref. 14. Briefly, 2 × 10^5^ HeLa cells were seeded in 6-well plates (Thermo Scientific) containing 22 × 22 mm glass coverslips a day before infection. HeLa cells were washed with phosphate-buffered saline (PBS) pH 7.4 and 1 mL of DMEM (without FBS) was added to each well before infection. Bacterial cultures were grown as described and cell cultures were infected with *Shigella* strains at a multiplicity of infection (MOI) of 100. After infection, cells were centrifuged at 110× g for 10 min at room temperature and plates were left for incubation at 37 °C with 5% CO_2_ for 30 min. Infected cultures were washed three times with PBS (pH 7.4) and further incubated with 2 mL of fresh DMEM containing 10% FBS and 50 μg/mL gentamicin at 37 °C with 5% CO_2_ for 3 h.

*Salmonella* infections were adapted from ref. 40. Briefly, 2 × 10^5^ HeLa cells were seeded in 6-well plates (Thermo Scientific) containing 22 × 22 mm glass coverslips a day before infection. HeLa cells were washed with phosphate-buffered saline (PBS) pH 7.4 and 1 mL of DMEM (without FBS) was added to each well before infection. Bacterial cultures were grown as described and cell cultures were infected with *Salmonella* strains at a multiplicity of infection (MOI) of 50. After infection, plates were left for incubation at 37 °C with 5% CO_2_ for 30 min. Infected cultures were washed three times with PBS (pH 7.4) and further incubated with 2 mL of fresh DMEM containing 10% FBS and 50 μg/mL gentamicin at 37 °C with 5% CO_2_ for 3 h. Cells were washed three times with PBS and fixed in 4% paraformaldehyde (PFA) for 15 min at room temperature for immunofluorescence.

### Immunofluorescence.

At 3 h 40 min post infection, cells were washed three times with PBS and fixed in 4% paraformaldehyde (PFA) for 15 min at room temperature. They were then washed again with PBS, permeabilized for 5 min with 0.1% Triton X-100 in PBS and incubated for 1 h with primary and secondary antibodies, each diluted in PBS containing 0.1% Triton X-100 and 1% bovine serum albumin. For samples requiring DNA staining, Hoechst was included along with the secondary antibody. Finally, coverslips were washed three times with PBS and mounted on glass slides using ProLong™ Diamond Antifade Mountant (Invitrogen, P36970I).

### Quantifying Intracellular Septin Recruitment.

Workflow to quantify the proportion of intracellular bacteria in septin cages were adopted from ref. 14. In brief, we used PFA-fixed samples stained with Hoechst and an anti-SEPT7 antibody. Image acquisition was performed as z-stacks spanning 3 µm with 185 nm interval. Using the Cell Counter plug-in in Fiji, we first determined the total number of bacteria in each field of view, then identified those exhibiting septin recruitment on their surface. Experimental datasets were anonymized prior to analysis. The percentage of septin-bound bacteria was calculated by dividing the number of septin-positive bacteria by the total bacterial count.

### Intracellular Replication Assay.

For intracellular replication experiments, *Shigella* infections were performed as described (*Human Cell Tissue Culture and Infections*). At indicated time points, 0.1% Triton X-100 (in PBS) was used for cell lysis and release of intracellular bacteria. Serial dilutions of each bacterial suspension were plated on TSB-agar plates and colony-forming units (CFUs) were determined at 16 h post incubation at 37 °C. CFU ratio (*t*_2_/*t*_1_) was calculated by dividing the number of colonies observed at 3 h 40 min (*t*_2_) post infection by 1 h 40 min (*t*_1_) post infection.

### siRNA Transfection.

Workflow for depleting SEPT7 in HeLa cells were adapted from ref. 52. We chose to deplete SEPT7 because it is widely recognized that SEPT7 controls septin hexamer and octamer function (14). Briefly, HeLa cells were transfected either using control (*mock*) siRNA or siRNA targeting SEPT7. For transfection, oligofectamine was used as instructed by the manufacturer. Cells were further incubated for 72 h to ensure sufficient knockdown of targeted proteins.

### Western Blotting.

Protein gels were transferred to 0.2 μm polyvinylidene difluoride membranes (PVDF, Biorad). PVDF membranes were then subjected to Ponceau S staining for 10 min at room temperature. Membranes were then destained with water until protein bands were visible on PVDF membrane. PVDF membranes were then incubated in blocking buffer (TBST buffer containing 5% of Milk) for 1 h. PVDF membranes were incubated with primary antibodies diluted in TBST overnight at 4 °C. PVDF membranes were washed three times for 10 min in TBST at room temperature and incubated with secondary goat HRP-conjugated antibodies in blocking buffer for 2 h at room temperature. Membranes were developed using PierceTM ECL plus western blotting substrate (Thermo Scientific).

### Flow Cytometry.

*Shigella* (2.5 × 10^4^ single cells) were analyzed by flow cytometry with an LSRII (BD Biosciences) flow cytometer. Data were analyzed using FlowJo software version 10.9.0. The mean fluorescence of each replicate was determined and normalized by its corresponding control condition.

## Supplementary Material

Appendix 01 (PDF)

Movie S1. A representative SMLM reconstructed 3D stack showing a group of *Shigella* ΔrfaC cells after in vitro septin-binding assay with WT octamers. Examples of visible septin polymers are shown with green arrows.

Movie S2. A representative SMLM reconstructed 3D stack showing a group of *Shigella* ΔrfaC cells after in vitro septin-binding assay with mutant octamers (SEPT2NCmut).

## Data Availability

DESeq2 lists from RNA sequencing analysis have been included in the *SI Appendix*. Matlab codes used for data analysis are deposited in GitHub (53).
