# Supplementary material for: Septin binding restricts Shigella growth and triggers intracellular tolerance
Source: Proc Natl Acad Sci U S A. 2026 Oct 27;123(44):e2529720123. doi: 10.1073/pnas.2529720123 (PMC13732209; doi:10.1073/pnas.2529720123)
Supplement: Supplementary file 1 [file pnas.2529720123.sapp.pdf]

Supporting Tables

Table S1. List of strains, plasmids and primers used in this study.

**Strains**	**Relevant genotype**	**Origin**
Shigella flexneri srv. 5a str. M90T	M90T, wild type	(1)
S. flexneri Δ*rfaL*	Δ*rfaL*	(2)
S. flexneri Δ*galU*	Δ*galU*	(2)
S. flexneri Δ*rfaC*	Δ*rfaC*	(2)
S. flexneri Δ*ospC1*	Δ*ospC1*	Gift from Felix Randow
S. flexneri Δ*cspC*	*cspC::aph (Kan)*	This study
Escherichia coli DH5a	DH5a, wild type	Thermo Fisher (#18265017)
E. coli BL21 (DE3)	BL21, wild type	Thermo Fisher (#EC0114)
Salmonella enterica serovar Typhimurium	NCTC 12023, wild type	Gift from Felix Randow
Salmonella Δ*rfaJ*	*rfaJ* $^-$	Gift from Felix Randow
Salmonella mCherry	Constitutively producing mCherry, (Carb^R)	Gift from Felix Randow
Salmonella picsA	Plasmid encoding and constitutively expressing *icsA*, Carb^R	This study
Salmonella Δ*rfaJ* picsA	*RfaJ* $^-$, Plasmid encoding and constitutively expressing *icsA*, Carb^R	This study

**Plasmids**	**Relevant genotype**	**Origin**
pKD46	*repA101*(ts) *oriR101* bla *ParaB*I-Red recombinase, Carb^R	(3)
pKD4	oriR$_{\gamma R6k}$ *FRT*::*kan*::*FRT*, Kan^R	(3)
pnEA-vH-SEPT2	P_{lac}-*SEPT2*	(4)
pnCS-msGFP–SEPT6-SEPT7	P_{lac}-*msGFP–SEPT6-SEPT7*	(4)
pmsGFP-SEPT6-HIS-SEPT2	P_{lac}-*msGFP–SEPT6-SEPT2*	(5)
pCMV_SEPT2-NCmut-mApple	*SEPT2NCmut-mApple*	(6)
pmsGFP-SEPT6-HIS-SEPT2NCmut	P_{lac}-*msGFP–SEPT6-SEPT2NCmut*	This study
pnCS_SEPT7_SEPT9_i1-TEV-Strep	P_{lac}-*SEPT7-SEPT9.i1*	(5)
picsA	Plasmid encoding and constitutively expressing *icsA*, Carb^R	(2)

Primers	Sequence (5’ to 3’)	Purpose
46_pmsGFP-SEPT6_SEPT2_fwd	TGAGGATCCTAATAGTCTAGAG	Gibson assembly
47_pmsGFP-SEPT6_SEPT2_rev	ATGGCCCTGGAAGTAAAG	Gibson assembly
48_SEPT2NCmut_fwd	acctttacttccagggccatATGTCTAAGCAACAACCAACTCAG	Gibson assembly
49_SEPT2NCmut_rev	ctagactattaggatcctcaCACATGGTGGCCGAGAGC	Gibson assembly
50_SEPT2	AACTGTCCAGATAGAGGCTTCGACTGTTG	Sequencing
72_SEPT2	CGCTCCTTCAGAGTGAGAGTGTCAG	Sequencing
139_cspC_del_fw	cgggcgaagaaaacaatctaaggaatttttcaaatgGTGTAGGCTGGAGCTGCTTC	cspC deletion
140_cspC_del_rv	aagcgtattcacacttcagatcagtggattcgatcagATATGAATATCCTCCTTAG	cspC deletion
140_cspC_checkDel_fw	caggatactaaaggccgcaa	Confirmation of cspC deletion
141_cspC_checkDel_rv	ggttaaacgaggtacagttctg	Confirmation of cspC deletion
200_gyrB_fw	GGCATCTGACGATAGAAGAAGG	qRT-PCR
201_gyrB_rv	AGTACAACCCGGACAAACTG	qRT-PCR
202_kdpB_fw	CTGCCGATCCAGACGATAAA	qRT-PCR
203_kdpB_rv	CGTAAACAACTGGCGCTATTC	qRT-PCR
206_cspC_fw	CGAACTCAACGTTCTGACCT	qRT-PCR
207_cspC_rv	GCTTCATTACTCCGGCTGAT	qRT-PCR
208_glnH_fw	GCACGATACGCCAAACATTC	qRT-PCR
209_glnH_rv	CTACCTTTCGGGAACGCAATA	qRT-PCR

Table S2. Results of DESeq2 differential expression analysis comparing control and septin-treated transcriptomes. Displayed are genes with adjusted *p*-values < 0.1.

**ID**	**Gene name**	**Base Mean**	**log2(Fold Change)**	**lfcSE**	**stat**	***p*-value**	**Adjusted *p*-value**
SF0599	*kdpB*	402.85	1.52	0.28	-5.44	5.4E-08	0.0002
SF3943	*yihM*	242.76	1.57	0.34	-4.63	3.6E-06	0.0067
SF0598	*kdpA*	445.29	1.19	0.27	-4.44	9.1E-06	0.0113
SF3158	*tdcA*	99.24	2.15	0.51	-4.23	2.3E-05	0.0218
SF3944	*yihN*	35.85	1.92	0.47	-4.12	3.8E-05	0.0286
SF2316	*nrdA*	1009.41	-0.89	0.22	4.03	5.6E-05	0.0347
SF2961	*nupG*	1124.93	1.13	0.29	-3.92	8.8E-05	0.0439
SF3939	*glnL*	728.10	-1.11	0.29	3.89	1.0E-04	0.0439
SF3942	*yihL*	198.99	1.15	0.30	-3.88	1.1E-04	0.0439
SF0258	*ykgG*	299.38	1.65	0.43	-3.81	1.4E-04	0.0486
SF0760	*glnQ*	1487.05	-1.11	0.29	3.77	1.6E-04	0.0486
SF0761	*glnP*	761.10	-1.18	0.31	3.77	1.6E-04	0.0486
SF_p0094	*ospC1*	333.27	1.61	0.43	-3.76	1.7E-04	0.0486
SF_p0079	*ipaH4.5*	677.72	1.73	0.47	-3.67	2.5E-04	0.0631
SF0630	*ybeK*	419.39	1.01	0.28	-3.66	2.5E-04	0.0631
SF2891	*gcvT*	2881.81	1.02	0.28	-3.61	3.0E-04	0.0707
SF4556	*micF*	126.78	2.19	0.61	-3.60	3.2E-04	0.0707
SF1584	*ydeY*	148.21	1.28	0.36	-3.53	4.2E-04	0.0878
SF0259	*ykgF*	990.46	1.63	0.46	-3.51	4.5E-04	0.0890
SF0762	*glnH*	5075.82	-0.88	0.25	3.48	5.0E-04	0.0933
SF1403	*cspC*	6829.61	1.00	0.29	-3.45	5.6E-04	0.0990
SF1583	*lsrA*	432.32	1.26	0.37	-3.44	5.9E-04	0.0996

Table S3. Results of DESeq2 differential expression analysis comparing control and septin-treated transcriptomes. Displayed are all virulence genes expressed from T3SS.

**ID**	**Gene name**	***p*-value**	**log2(fold change)**
SF_p0094	*ospC1*	0.000	1.6
SF_p0079	*ipaH4.5*	0.000	1.7
SF_p0078	*ipaH7.8*	0.001	1.7
SF_p0003	*ospB*	0.006	1.3
SF_p0226	*ipaH9.8*	0.007	1.3
SF_p0082	*ipgH*	0.007	1.0
SF_p0005	*ospC4*	0.008	1.4
SF_p0048	*ospE2*	0.009	1.3
SF_p0181	*virA*	0.009	1.1
SF_p0227	*ospG*	0.012	1.1
SF_p0265a	*ospE1*	0.015	1.3
SF_p0010	*ospF*	0.015	0.9
SF_p0141	*mxiN*	0.016	1.3
SF_p0063	*ospC2*	0.017	1.4
SF_p0132	*icsB*	0.023	0.9
SF_p0131	*ipgA*	0.030	2.1
SF_p0134	*ipgE*	0.034	1.0
SF_p0137	*mxiH*	0.036	1.1
SF_p0135	*ipgF*	0.037	1.1
SF_p0093	*ospD3*	0.038	0.6
SF_p0130	*ipgB1*	0.043	0.8
SF_p0140	*mxiK*	0.046	1.0
SF_p0054	*ipaH2.5*	0.047	1.4
SF_p0155	*spa29*	0.048	1.1
SF_p0152	*spa33*	0.050	1.1
SF_p0139	*mxiJ*	0.059	0.8
SF_p0136	*mxiG*	0.062	0.9
SF_p0143	*mxiM*	0.066	1.8
SF_p0237	*virK*	0.073	0.5
SF_p0147	*mxiA*	0.073	0.9
SF_p0142	*mxiL*	0.073	1.6
SF_p0115	*ospC3*	0.074	0.9
SF_p0154	*spa9*	0.076	1.0
SF_p0133	*ipgD*	0.076	0.8
SF_p0122	*ipaJ*	0.079	0.7
SF_p0148	*spa15*	0.079	0.8
SF_p0024	*ipgB2*	0.083	0.9

SF_p0156	*spa40*	0.086	1.0
SF_p0138	*mxiI*	0.090	0.7
SF_p0153	*spaP*	0.091	0.8
SF_p0265	*ipaH1.4*	0.111	0.6
SF_p0271	*icsP/sopA*	0.132	0.6
SF_p0146	*mxiC*	0.137	0.6
SF_p0046	*virF*	0.143	0.8
SF_p0145	*mxiD*	0.145	0.6
SF_p0151	*spa32*	0.189	0.6
SF_p0182	*icsA/virG*	0.346	0.3
SF_p0125	*ipaA*	0.360	0.4
SF_p0126	*ipaD*	0.380	0.3
SF_p0009	*ospD2*	0.385	0.3
SF_p0144	*mxiE*	0.396	0.3
SF_p0123	*virB*	0.399	0.3
SF_p0150	*spa13*	0.446	0.3
SF_p0129	*ipgC*	0.489	0.2
SF_p0127	*ipaC*	0.646	0.1
SF_p0149	*spa47*	0.695	0.1
SF_p0128	*ipaB*	0.731	-0.1

Table S4. Results of DESeq2 differential expression analysis comparing control and Congo red treated transcriptomes. Displayed are all virulence genes expressed from T3SS.

**ID**	**Gene name**	***p*-value**	**log2(fold change)**
SF_p0131	*ipgA*	3.66E-06	2.2
SF_p0063	*ospC2*	3.04E-05	1.5
SF_p0134	*ipgE*	4.90E-05	1.4
SF_p0137	*mxiH*	5.21E-05	1.3
SF_p0141	*mxiN*	5.04E-05	1.6
SF_p0130	*ipgB1*	8.88E-05	1.0
SF_p0005	*ospC4*	0.000	1.3
SF_p0142	*mxiL*	0.000	1.8
SF_p0143	*mxiM*	0.000	2.0
SF_p0135	*ipgF*	0.000	1.3
SF_p0136	*mxiG*	0.000	1.1
SF_p0094	*ospC1*	0.001	1.2
SF_p0153	*spaP*	0.001	1.1
SF_p0152	*spa33*	0.001	1.4
SF_p0140	*mxiK*	0.002	1.1
SF_p0133	*ipgD*	0.002	1.0
SF_p0139	*mxiJ*	0.002	0.9
SF_p0148	*spa15*	0.002	1.0
SF_p0147	*mxiA*	0.003	1.1
SF_p0138	*mxiI*	0.003	0.8
SF_p0046	*virF*	0.005	1.0
SF_p0146	*mxiC*	0.006	0.9
SF_p0154	*spa9*	0.006	1.2
SF_p0227	*ospG*	0.007	0.9
SF_p0155	*spa29*	0.009	1.1
SF_p0010	*ospF*	0.009	0.7
SF_p0127	*ipaC*	0.010	0.5
SF_p0122	*ipaJ*	0.011	0.7
SF_p0151	*spa32*	0.012	0.9
SF_p0003	*ospB*	0.012	0.8
SF_p0024	*ipgB2*	0.012	0.9
SF_p0181	*virA*	0.013	0.7
SF_p0145	*mxiD*	0.017	0.7
SF_p0156	*spa40*	0.018	0.9
SF_p0082	*ipgH*	0.020	0.5
SF_p0115	*ospC3*	0.030	0.7
SF_p0129	*ipgC*	0.034	0.5

SF_p0271	*icsP/sopA*	0.034	0.7
SF_p0132	*icsB*	0.035	0.6
SF_p0126	*ipaD*	0.057	0.5
SF_p0125	*ipaA*	0.065	0.6
SF_p0150	*spa13*	0.069	0.5
SF_p0182	*icsA/virG*	0.097	0.4
SF_p0144	*mxiE*	0.106	0.4
SF_p0009	*ospD2*	0.110	0.4
SF_p0048	*ospE2*	0.171	0.5
SF_p0265a	*ospE1*	0.172	0.6
SF_p0149	*spa47*	0.213	0.3
SF_p0123	*virB*	0.229	0.2
SF_p0093	*ospD3*	0.255	0.2
SF_p0128	*ipaB*	0.294	0.3
SF_p0079	*ipaH4.5*	0.341	0.4
SF_p0078	*ipaH7.8*	0.576	0.4
SF_p0237	*virK*	0.727	0.1
SF_p0226	*ipaH9.8*	0.940	0.1
SF_p0265	*ipaH1.4*	0.886	0.1
SF_p0054	*ipaH2.5*	0.093	1.2

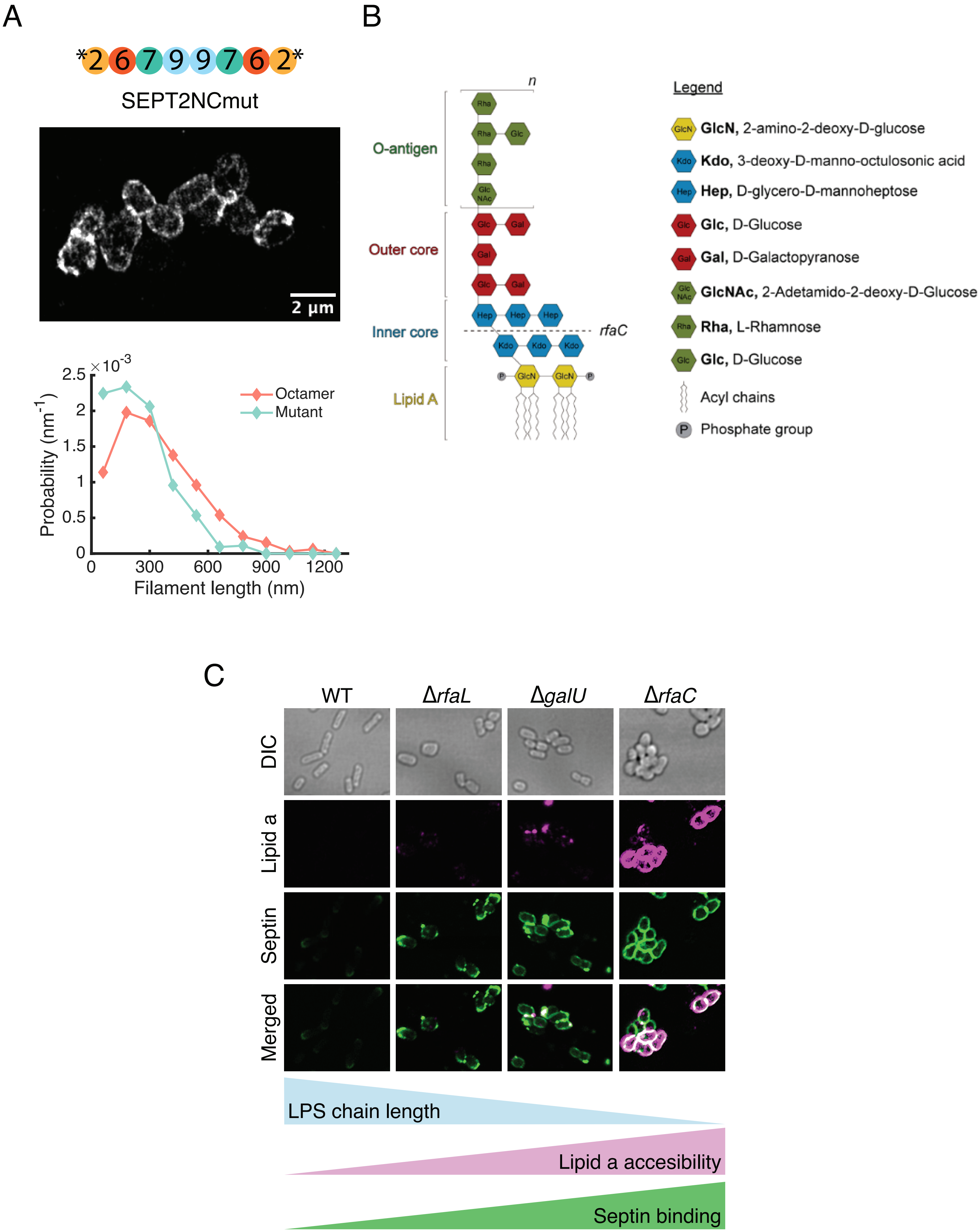

A
2 6 7 9 9 7 6 2
SEPT2NCmut
2 µm
×10-3
2.5
2
1.5
1
0.5
0
Probability (nm-1)
0
300
600
900
1200
Filament length (nm)
Octamer
Mutant
B
n
O-antigen
Outer core
Inner core
Lipid A
rfaC
Rha
Glc
GlcNAc
Gal
Hep
Kdo
GlcN
P
Legend
GlcN, 2-amino-2-deoxy-D-glucose
Kdo, 3-deoxy-D-manno-octulosonic acid
Hep, D-glycero-D-mannoheptose
Glc, D-Glucose
Gal, D-Galactopyranose
GlcNAc, 2-Adetamido-2-deoxy-D-Glucose
Rha, L-Rhamnose
Glc, D-Glucose
Acyl chains
Phosphate group
C
WT
ΔrfaL
ΔgalU
ΔrfaC
DIC
Lipid a
Septin
Merged
LPS chain length
Lipid a accesibility
Septin binding

Figure S1

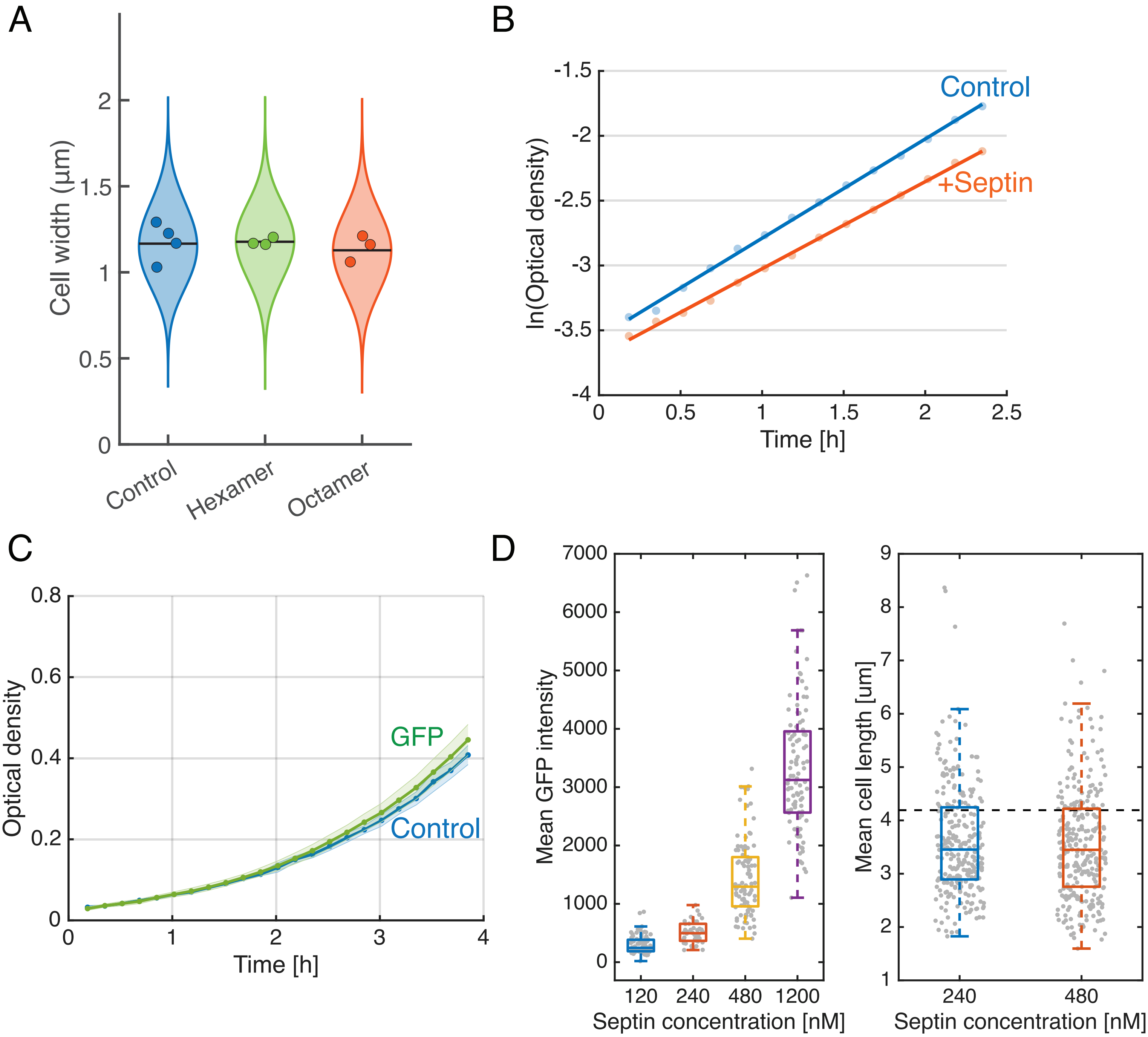

Figure S2

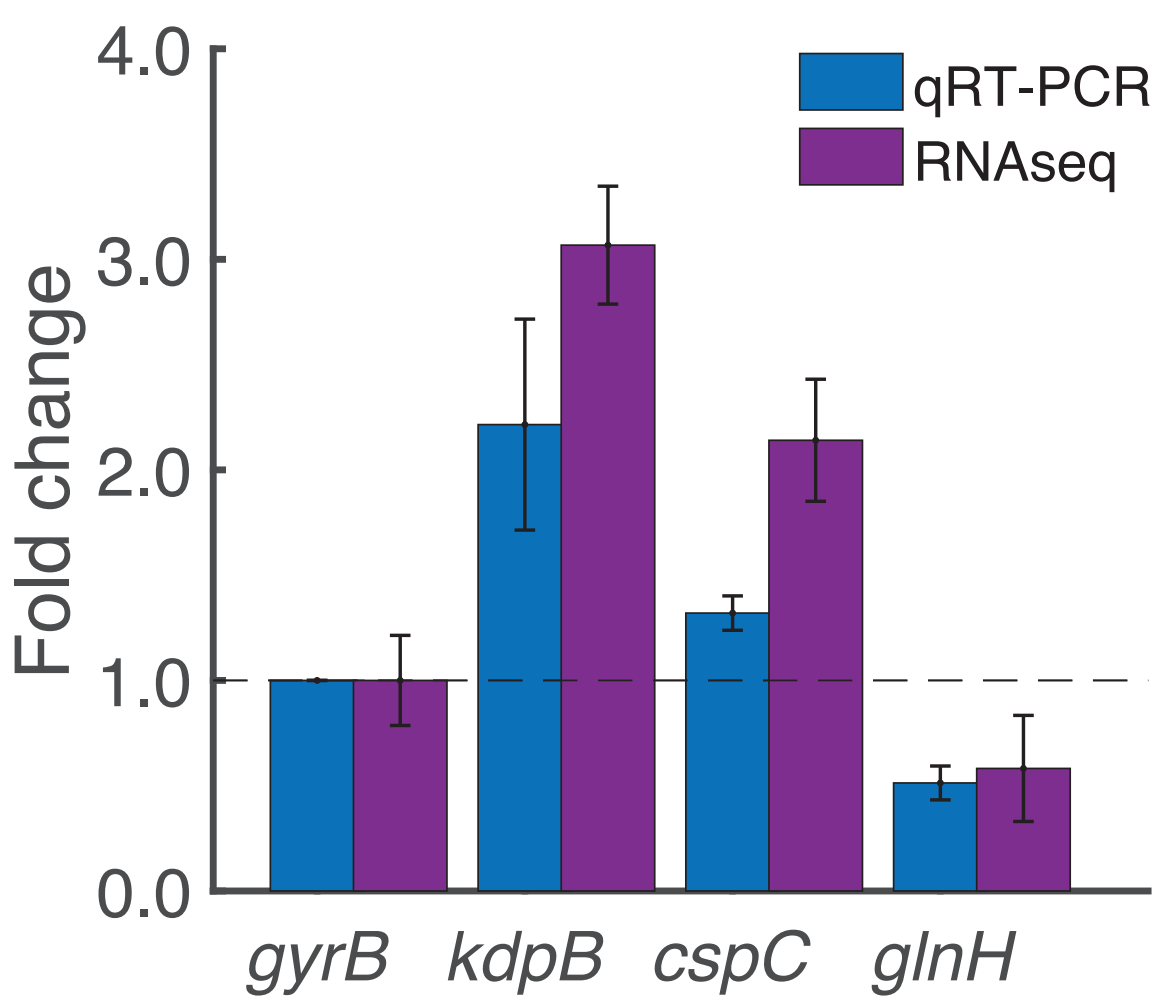

Figure S3

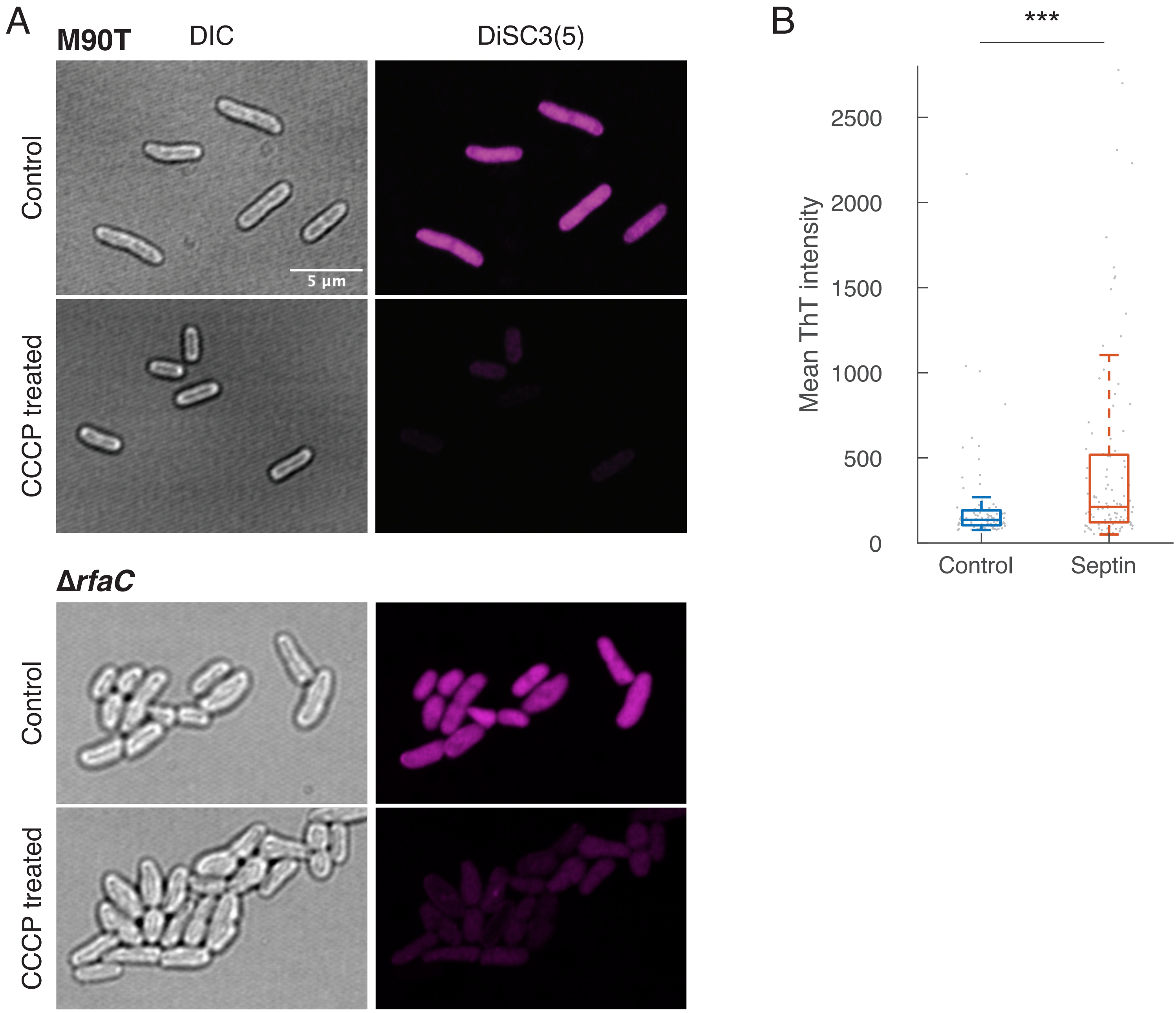
A
M90T
DIC
DiSC3(5)
Control
CCCP treated
5 µm
ΔrfaC
Control
CCCP treated
B
***
Mean ThT intensity
2500
2000
1500
1000
500
0
Control
Septin

Figure S4

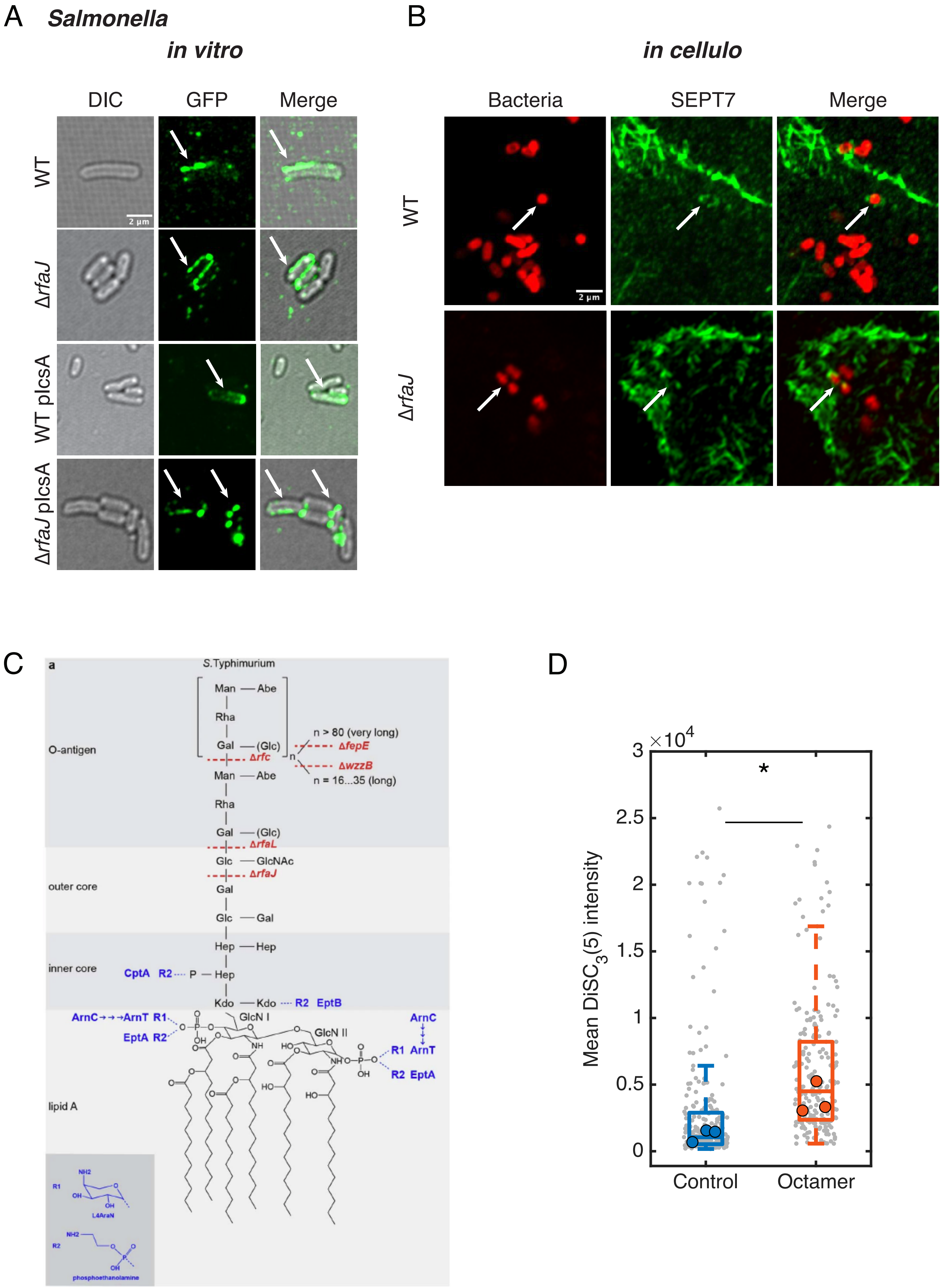
A
Salmonella
in vitro
DIC
GFP
Merge
WT
ΔrfaJ
WT pIcsA
ΔrfaJ pIcsA
2 µm
B
in cellulo
Bacteria
SEPT7
Merge
WT
ΔrfaJ
2 µm
C
a
S.Typhimurium
O-antigen
Man
Abe
Rha
Gal
(Glc)
n
n > 80 (very long)
ΔfepE
Δrfc
ΔwzzB
n = 16...35 (long)
ΔrfaL
outer core
Glc
GlcNAc
ΔrfaJ
Gal
inner core
Hep
CptA R2
P
Kdo
R2 EptB
ArnC
ArnT R1
EptA R2
GlcN I
GlcN II
OH
NH
HO
R1 ArnT
R2 EptA
lipid A
R1
NH2
L4AraN
R2
phosphoethanolamine
D
3
×10^4
2.5
2
1.5
1
0.5
0
*
Mean DiSC3(5) intensity
Control
Octamer

Figure S5

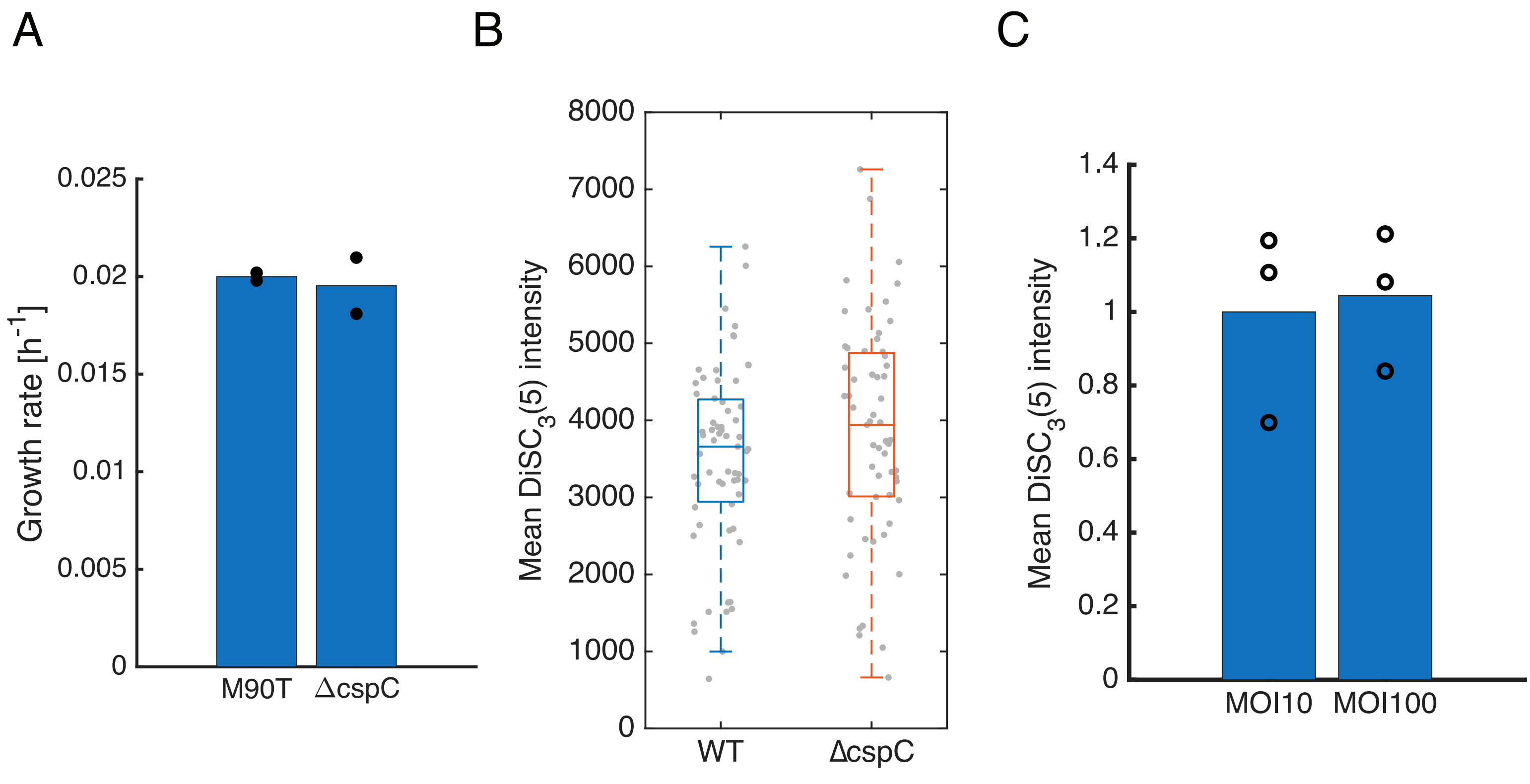

A
B
C
Growth rate [h^-1]
0.025
0.02
0.015
0.01
0.005
0
M90T
ΔcspC
Mean DiSC3(5) intensity
8000
7000
6000
5000
4000
3000
2000
1000
0
WT
ΔcspC
Mean DiSC3(5) intensity
1.4
1.2
1
0.8
0.6
0.4
0.2
0
MOI10
MOI100

Figure S6

Supporting Figure Legends

Figure S1. Septin binding increases with increased lipid A accessibility

A. Top. Septin interface mutant (SEPT2NCmut) carries point mutations in the SEPT2-SEPT2 NC interface, disabling oligomer-oligomer interactions. A representative SMLM reconstructed image showing a group of *Shigella* cells after *in vitro* septin-binding assay with no visible septin polymers. Bottom. Probability density distribution of filament lengths of WT and mutant septin octamers.

B. A cartoon showing bacterial LPS of *S. flexneri* 5a. Dashed line indicates the truncation of the O-antigen in Δ*rfaC* mutant. Adapted from (2).

C. Representative DIC and fluorescence microscopy images of *Shigella* wild type and LPS mutants (Δ*rfaL*, Δ*galU*, Δ*rfaC*). From left to right, the mutants display progressively shorter LPS chain lengths. Lipid A labeling with Alexa Fluor 647 shows increased fluorescence intensity as LPS chain length decreases. Similarly, septin (GFP-tagged) intensity increases in parallel with lipid A labeling.

Figure S2. Septin binding inhibits growth of *Shigella*

A. Violin plots show probability distributions of single cell width measurements of *Shigella* Δ*rfaC* which are collected from different biological replicates. Mean of each biological replicate is shown as darker shade circles with black outline. Minimum 3 biological replicates per condition. Pairwise comparisons to control do not show significant differences.

B. Natural logarithm of growth curves showing exponential phase of control and septin-exposed *Shigella ΔrfaC.*

C. Growth curves at exponential phase of control and GFP-exposed *Shigella ΔrfaC.*

D. Left. Mean GFP intensity on the bacterial (*Shigella ΔrfaC)* surface under different concentrations of septins. Right. Boxplots of single cell lengths of septin-bound *Shigella* Δ*rfaC*. Dashed black line shows the bacterial length of untreated control as reference.

Figure S3. qRT-PCR fold changes in comparison to RNA-sequencing

qRT-PCR analysis of selected genes confirmed expression changes detected by RNA-sequencing. Bars represent relative fold changes in expression (mean ± standard deviation of 3 biological replicates), plotted alongside corresponding fold changes obtained from RNA-sequencing analysis. *gyrB* is used as the reference gene.

Figure S4. CCCP treatment leads to rapid depolarization of bacterial membrane

A. A group of *Shigella* (M90T and Δ*rfaC*) cells before and after CCCP treatment (10 µg/ml) for 5 minutes. Representative images of cells with (Left) DIC and (Right) DiSC3(5) signal showing rapid depolarization and loss of signal upon CCCP treatment, as expected.

B. Boxplots of single cell mean ThT intensity values of control and septin-bound samples (*Shigella* ∆*rfaC*) shown. Gray circles represent single cell values. Pairwise comparison of distributions by one-way ANOVA shows statistical significance with *p*-value < 0.0005.

Figure S5. Septins bind *Salmonella* in vitro and in cellulo

A. Representative images of septin-bound *Salmonella*. White arrows indicate areas of septin-binding to the bacterial surface.

B. Representative images of septin recruitment in HeLa cells infected with *Salmonella*, 3 hours 30 minutes post-infection. White arrows indicate septin-bound bacteria.

C. The composition of lipid A, inner core, outer core and O-antigen from *Salmonella*. Adapted from (7)

D. Septin binding causes hyperpolarization of *Salmonella* membrane. Single-cell mean DiSC3(5) intensity values are plotted as gray circles and median of biological replicates are shown as colored larger circles with black outline. Pairwise comparisons are made by comparing means of biological replicates. * shows $p<0.05$ according to Student's t-test. *Salmonella* ∆*rfaJ* picsA is used for this experiment.

Figure S6. Membrane potential changes of ∆*cspC* mutant during infection

Shigella M90T and ∆*cspC* mutant display similar **(A)** growth rates **(B)** and DiSC3(5) uptake in broth culture. Differences are non-significant.

C. Confirming bacterial load did not affect DiSC3(5) uptake by infecting HeLa cells with WT *Shigella* at multiplicities of infection (MOIs) of 10 and 100. Plot shows DiSC3(5) fluorescence levels measured by flow cytometry. Differences are non-significant.

Supporting Movie Captions

Movie 1. A representative SMLM reconstructed 3D stack showing a group of *Shigella* ∆*rfaC* cells after in vitro septin-binding assay with WT octamers. Examples of visible septin polymers are shown with green arrows.

Movie 2. A representative SMLM reconstructed 3D stack showing a group of *Shigella* ∆*rfaC* cells after in vitro septin-binding assay with mutant octamers (SEPT2NCmut).

References

1. S. Mostowy, *et al.*, Entrapment of Intracytosolic Bacteria by Septin Cage-like Structures. *Cell Host & Microbe* **8**, 433–444 (2010).

2. D. Lobato-Márquez, *et al.*, Mechanistic insight into bacterial entrapment by septin cage reconstitution. *Nat Commun* **12**, 4511 (2021).

3. K. A. Datsenko, B. L. Wanner, One-step inactivation of chromosomal genes in *Escherichia coli* K-12 using PCR products. *Proc. Natl. Acad. Sci. U.S.A.* **97**, 6640–6645 (2000).

4. M. Mavrakis, *et al.*, Septins promote F-actin ring formation by crosslinking actin filaments into curved bundles. *Nat Cell Biol* **16**, 322–334 (2014).

5. F. Iv, *et al.*, Insights into animal septins using recombinant human septin octamers with distinct SEPT9 isoforms. *Journal of Cell Science* **134**, jcs258484 (2021).

6. M. Kuzmić, *et al.*, Septin-microtubule association via a motif unique to isoform 1 of septin 9 tunes stress fibers. *Journal of Cell Science* **135**, jcs258850 (2022).

7. E. G. Otten, *et al.*, Ubiquitylation of lipopolysaccharide by RNF213 during bacterial infection. *Nature* **594**, 111–116 (2021).
